# Changes in Care Associated With Integrating Medicare and Medicaid for Dual-Eligible Individuals

**DOI:** 10.1001/jamahealthforum.2023.4583

**Published:** 2023-12-21

**Authors:** Eric T. Roberts, Lingshu Xue, John Lovelace, Chris Kypriotis, Kathryn L. Connor, Qingfeng Liang, David C. Grabowski

**Affiliations:** 1 Division of General Internal Medicine, University of Pennsylvania Perelman School of Medicine, Philadelphia; 2 Department of Health Policy and Management, University of Pittsburgh School of Public Health, Pittsburgh, Pennsylvania; 3 UPMC Insurance Services Division, Pittsburgh, Pennsylvania; 4 Center for High-Value Health Care, University of Pittsburgh Medical Center, Pittsburgh, Pennsylvania; 5 Department of Health Care Policy, Harvard Medical School, Boston, Massachusetts

## Abstract

**Importance:**

There is growing interest in expanding integrated models, in which 1 insurer manages Medicare and Medicaid spending for dually eligible individuals. Fully integrated dual-eligible special needs plans (FIDE-SNPs) are one of the largest integrated models, but evidence about their performance is limited.

**Objective:**

To evaluate changes in care associated with integrating Medicare and Medicaid coverage in a FIDE-SNP in Pennsylvania.

**Design, Setting, and Participants:**

This cohort study using a difference-in-differences analysis compared changes in care between 2 cohorts of dual-eligible individuals: (1) an integration cohort composed of Medicare Dual Eligible Special Needs Plan enrollees who joined a companion Medicaid plan following a 2018 state reform mandating Medicaid managed care (leading to integration), and (2) a comparison cohort with nonintegrated coverage before and after the start of Medicaid managed care. Analyses were conducted between February 2022 and June 2023.

**Main Outcomes and Measures:**

Analyses examined outcomes in 4 domains: use of home- and community-based services (HCBS), care management and coordination, hospital stays and postacute care, and long-term nursing home stays.

**Results:**

The study included 7967 individuals in the integration cohort and 3832 individuals in the comparison cohort. In the integration cohort, the mean (SD) age at baseline was 63.3 (14.7) years, and 5268 individuals (66.1%) were female and 2699 (33.9%) were male. In the comparison cohort, the mean (SD) age at baseline was 64.8 (18.6) years, and 2341 individuals (61.1%) were female and 1491 (38.9%) were male. At baseline, integration cohort members received a mean (SD) of 2.83 (8.70) days of HCBS per month and 3.34 (3.56) medications for chronic conditions per month, and the proportion with a follow-up outpatient visit after a hospital stay was 0.47. From baseline through 3 years after integration, HCBS use increased differentially in the integration vs comparison cohorts by 0.61 days/person-month (95% CI, 0.28-0.94; *P* < .001). However, integration was not associated with changes in care management and coordination, including medication use for chronic conditions (−0.02 fills/person-month; 95% CI, −0.10 to 0.06; *P* = .65) or follow-up outpatient care after a hospital stay (−0.01 visits/hospital stay; 95% CI, −0.04 to 0.03; *P* = .61). Hospital stays did not change differentially between the cohorts. Unmeasured factors contributing to differential mortality limited the ability to identify changes in long-term nursing home stays associated with integration.

**Conclusions and Relevance:**

In this cohort study with a difference-in-differences analysis of 2 cohorts of individuals dually eligible for Medicare and Medicaid, integration was associated with greater HCBS use but not with other changes in care patterns. The findings highlight opportunities to strengthen how integrated programs manage care and a need to further evaluate their performance.

## Key Points

**Question:**

Are integrated models, in which 1 insurer manages Medicare and Medicaid spending for dually eligible individuals, associated with improved care for this population?

**Findings:**

This difference-in-differences cohort study including 7967 individuals in the integration cohort and 3832 individuals in the comparison cohort found that the introduction of a fully integrated dual-eligible special needs plan in Pennsylvania was associated with increased use of home- and community-based services, but no changes in care coordination or hospitalizations, relative to changes in a comparison cohort maintaining nonintegrated coverage.

**Meaning:**

Integration was associated with increased use of community-based services and supports, but not with changes in other outcomes where there remains a need to improve the care of dually eligible individuals.

## Teaser

This cohort study evaluates changes in care associated with integrating Medicare and Medicaid coverage in a fully integrated dual-eligible special needs plan in Pennsylvania.

## Introduction

Policymakers are testing new models of integrating insurance coverage for dual-eligible individuals (ie, people eligible for and enrolled in Medicare and Medicaid) to improve care for this population.^1,2,3,4^ Dual-eligible individuals often have complex care needs but face challenges receiving high-quality and coordinated care because they navigate 2 separate insurance programs for care.^5,6,7^ Specifically, Medicare (a federal program) is the primary payer for hospital and postacute care, outpatient care, and prescription drugs, while Medicaid (managed by states) pays for long-term care, including nursing home care and home- and community-based services (HCBS).^8^ Concerns that bifurcated coverage contributes to fragmented and inefficient care have led policymakers to test integrated programs in which 1 organization, such as a managed care plan, coordinates care and manages Medicare and Medicaid spending for the same people.^2,9^

Fully integrated dual-eligible special needs plans (FIDE-SNPs) are among the largest integrated programs, with approximately 400 000 enrollees across 66 plans in 2023.^10^ FIDE-SNPs are a subset of dual-eligible special needs plans (D-SNPs), which are Medicare Advantage plans that exclusively serve dual-eligible individuals.^11^ A distinguishing feature of FIDE-SNPs is that these plans have capitation contracts to manage Medicaid long-term care spending (80% of Medicaid spending among dual-eligible individuals).^4,12^ In some states, FIDE-SNPs also manage Medicaid behavioral health spending.^13^ These arrangements result in the integration of Medicare and Medicaid coverage within 1 managed care plan.^1,3^

Conceptually, integration creates incentives to deliver care efficiently, because 1 plan internalizes all costs of care.^2^ For example, FIDE-SNPs may enhance the provision of HCBS to manage enrollees’ chronic and disabling health conditions, as doing so may help to avoid costly hospital and nursing home admissions linked to unmet care needs.^14,15^ Further, because FIDE-SNPs manage Medicare and Medicaid services for the same people, the plans may be equipped to identify enrollees who need supportive services.

Evidence remains limited as to whether FIDE-SNPs improve care for dual-eligible individuals.^16^ Prior evaluations, including the few that examined FIDE-SNPs, found that integration was associated with increased HCBS use,^9,17,18^ fewer nursing home admissions in several (though not all) models,^2,9,19,20,21,22,23^ and variable changes in hospitalizations.^2,19,20,21^ However, because enrollment in FIDE-SNPs (like all Medicare Advantage plans) is voluntary, unmeasured differences between enrollees in these vs other plans may bias evaluations. A recent review concluded that evaluations of integrated programs often did not adequately address bias, and it called for greater attention to this issue in future studies.^16^

This study examined changes in care associated with integrating Medicare and Medicaid in a FIDE-SNP run by UPMC for You (hereafter, UPMC), an insurer in Pennsylvania. In 2023, UPMC’s FIDE-SNP served approximately 39 000 dual-eligible individuals, making it the largest FIDE-SNP in the US by enrollment.^10^ This integrated plan formed following a 2018 reform in which Pennsylvania established a mandatory Medicaid managed care program for older adults and disabled individuals (most of whom are dual-eligible individuals) and provided opportunities to integrate coverage through companion Medicare and Medicaid plans run by the same insurers. We used a difference-in-differences design to compare changes in care between 2 cohorts of dual-eligible individuals: 1 cohort that received integrated coverage through companion UPMC plans starting in 2018 and a comparison cohort that maintained nonintegrated coverage. We also examined limitations of this design in controlling for unmeasured factors correlated with outcome trends, which bias difference-in-differences analyses.^24^

## Methods

### Study Context

We studied a FIDE-SNP that formed after the introduction of Community HealthChoices (CHC), a mandatory Medicaid managed care program for older adults and disabled individuals in Pennsylvania.^25^ CHC replaced a fee-for-service Medicaid program, under which there was little opportunity to integrate Medicaid and Medicare services for dual-eligible individuals.

Under CHC, 3 insurers, including UPMC, operated statewide Medicaid managed care plans. These insurers were also required to operate companion D-SNPs, coordinate Medicaid and Medicare services, and assign enrollees to same primary care clinicians in D-SNPs and Medicaid plans (for enrollees of companion plans).^25,26^ These changes increased opportunities to integrate coverage, particularly for D-SNP enrollees.

CHC was introduced in phases beginning in January 2018 in 14 counties in southwestern Pennsylvania, where UPMC has operated a D-SNP since 2013. Approximately 85% of existing enrollees in UPMC’s D-SNP joined its companion Medicaid plan in 2018 (eFigure 1 in Supplement 1). This change meant that UPMC began to manage Medicare and Medicaid spending for enrollees of companion plans, prompting the insurer to apply for FIDE-SNP designation starting in 2018. Although this designation was not finalized until 2020, we analyzed 2018 to 2020 as the postintegration period because the key element of an integrated plan—1 entity managing Medicare and Medicaid spending—was present over this period. UPMC’s FIDE-SNP covered Medicaid long-term care spending, Medicare cost sharing, and some services that Medicare does not cover. However, it did not cover Medicaid-funded behavioral health spending, which is managed separately from CHC.^25^

Although dual-eligible individuals were required to enroll in Medicaid managed care, enrollment in Medicare Advantage (including D-SNPs) remained voluntary. We included dual-eligible individuals in southwestern Pennsylvania with continuous enrollment in traditional Medicare (while alive) as a comparison cohort that maintained nonintegrated coverage. The comparison cohort controls for changes in outcomes unrelated to integration, including changes linked to the introduction of Medicaid managed care, which affected both cohorts.

### Data

We analyzed data from 3 main sources. First, we analyzed UPMC Medicare enrollment and claims data from 2015 to 2020 and UPMC Medicaid data from 2018 to 2020. Second, we analyzed Medicaid service history files, which reported 2017 claims from Pennsylvania’s prior fee-for-service Medicaid program. The files also reported individual-level enrollment in HCBS waiver programs and receipt of long-term nursing home care in 2017. Third, we obtained 2015 to 2020 Medicare enrollment and claims for a comparison cohort of dual-eligible individuals with traditional Medicare. eFigure 2 in Supplement 1 illustrates file linkages. The University of Pittsburgh Institutional Review Board approved this study and granted a waiver of informed consent because this study used deidentified secondary data.

### Study Cohorts

The integration cohort included dual-eligible individuals in UPMC’s D-SNP in the baseline period (2015-2017) who remained enrolled, while alive, in UPMC’s D-SNP and Medicaid managed care plan in the postperiod (2018-2020). The comparison cohort included dual-eligible individuals with traditional Medicare at baseline who remained enrolled, while alive, in traditional Medicare and UPMC’s Medicaid managed care plan in the postperiod. Both cohorts were limited to dual-eligible individuals with full Medicaid. We followed Strengthening the Reporting of Observational Studies in Epidemiology (STROBE) reporting guideline for cohort studies.

Individuals who died in the postperiod were included while they remained alive. However, the inclusion criteria required individuals to have survived throughout the baseline period (until January 2018) to be able to join UPMC’s Medicaid managed care plan. Differential attrition from mortality in the postperiod may affect our estimates, particularly in contexts where mortality is high (eg, among nursing home residents). We examined this issue in a sensitivity analysis.

### Outcomes

We analyzed 4 sets of person-month–level outcomes. First, we analyzed days of HCBS use, which include personal care services that assist people with activities of daily living (eg, eating, bathing, and dressing), rehabilitation, and therapy services. These services are covered by Medicaid and are typically provided on a long-term basis to people with a disability.^27^ Second, we analyzed several care management and coordination measures: outpatient visits; standardized 30-day fills of medications for chronic conditions; and proportion of hospital visits (inpatient admissions, emergency department visits, and observation stays) followed within 14 days by an outpatient visit.^28^ Third, we analyzed days of inpatient care, inpatient admissions for ambulatory care–sensitive conditions,^29^ and emergency department visits and observation stays. We also analyzed days of Medicare-covered skilled nursing facility care and home health care (ie, home-based postacute care). Fourth, we analyzed days of long-term nursing home care covered by Medicaid.^9,21^ The eAppendix in Supplement 1 reports variable definitions.

### Covariates

We included the following covariates: age in 2017, sex, race and ethnicity, and disability (original reason for Medicare entitlement). Race and ethnicity were measured from administrative data. To protect confidentiality, racial and ethnic groups with smaller representation in our sample (Asian or Pacific Islander, American Indian or Alaska Native, or another race) were reported as a single other group. However, separate indicators for each racial and ethnic group were included in regression analyses. We used zip codes in 2017 to identify residents of rural communities and to include the Area Deprivation Index.^30^ We used diagnoses in 2017 Medicare inpatient, outpatient, and professional claims to construct an Elixhauser Comorbidity Index^31^ that characterized chronic disease burden at baseline.

### Statistical Analyses

For each outcome, we estimated a linear regression model (with the person-month as the unit of analysis), where the main explanatory variables were an indicator for membership in the integration cohort; indicators for 2018, 2019, and 2020; and interactions between integration cohort and year. Coefficients on these interactions are our difference-in-differences estimates and represent differential changes between the integration vs comparison cohorts from baseline through the first, second, and third years after integration. We estimated changes through each postyear to account for evolving care patterns as the integrated plan matured and eventually became designated as a FIDE-SNP. This also enabled us to estimate a separate effect for being in an integrated plan in 2020 to account for disruptions in care from steady state trends due to COVID-19.

We adjusted for covariates described above and quarter and year fixed effects to account for temporal trends. Models were weighted using propensity score weights that balanced the cohorts on baseline covariates. Standard errors were clustered at the person level. The eAppendix in Supplement 1 provides additional information about our analyses.

#### Assumptions and Differential Attrition From Mortality

An assumption of difference-in-differences is that the comparison cohort controls for outcome trends that would have been expected without integration.^24^ We assessed the plausibility of this assumption by estimating event-study models to test whether outcome trends were parallel between the integration and comparison cohorts in the baseline period. However, a limitation of this test is that baseline outcome trends might appear parallel because analyses were limited to survivors over the baseline period. Unmeasured differences in mortality risk factors preceding integration could contribute to differential outcome trends beginning in 2018, but the influence of such factors would not be detected through a comparison of baseline trends.

We addressed this concern by analyzing differential outcome trends from baseline through year 1 of the postperiod (2018), before vs after excluding 2018 decedents from all periods. Although prior research identified plan effects on mortality,^32^ mortality differences after 1 year likely reflect unmeasured risk factors preceding integration. Appreciable changes in year-1 estimates when we exclude 2018 decedents would suggest that findings were sensitive to mortality differences early in the postperiod.

#### Supplementary Analyses

We conducted 3 supplementary analyses. First, to account for substantial baseline differences in long-term nursing home use between the cohorts—which may reflect differences in health, functional, and cognitive factors that influence long-run utilization trends—we ran analyses among a subset of dual-eligible individuals who lived in the community in 2017 (ie, excluding residents of nursing homes at baseline). Second, to further examine treatment effect heterogeneity among individuals with different baseline long-term care needs,^2^ we stratified analyses according to whether individuals in 2017 were enrolled in an HCBS waiver program (for people living in the community requiring a nursing home level of care), living in the community and not in an HCBS program, or long-term nursing home residents. Third, among community-dwelling individuals in 2017, we examined monthly changes in eligibility for long-term services (HCBS vs long-term nursing facility care). Although assessments were performed independently of insurers, insurers could make referrals for assessments based on enrollees’ health and functional status. These analyses elucidate how having 1 insurer manage Medicare and Medicaid services for the same people might have influenced referrals, and ultimately eligibility, for long-term care. Because we lacked monthly data prior to 2018, we descriptively characterized changes from 2018 to 2020. We compared results before and after excluding decedents from all periods.

We used 2-sided significance tests and considered *P* < .05 statistically significant. Analyses were conducted in Stata, version 16 (StataCorp LLC).

## Results

### Cohort Characteristics

At baseline, the integration cohort included 7967 individuals, and the comparison cohort included 3832 individuals (Table 1). In the integration cohort, the mean (SD) age in 2017 was 63.3 (14.7) years; 5268 individuals (66.1%) were female and 2699 (33.9%) were male; 1369 (17.2%) were non-Hispanic Black (hereafter, Black), 22 (0.3%) were Hispanic, 6360 (79.8%) were non-Hispanic White (hereafter, White), and 216 (2.7%) were of other race and ethnicity. In the comparison cohort, prior to propensity score weighting, the mean (SD) age in 2017 was 64.8 (18.6) years; 2341 individuals (61.1%) were female and 1491 (38.9%) were male; 347 (9.1%) were Black, 50 (1.3%) were Hispanic, 3325 (86.8%) were White, and 110 (2.9%) were of other race and ethnicity. Compared to integrated programs nationally, a smaller proportion of integration cohort members were Black or Hispanic.^33^ The mean (SD) Elixhauser Comorbidity Index at baseline was higher in the integration vs comparison cohorts (4.94 [3.28] vs 4.81 [3.59]). Conversely, the proportion of long-term nursing home residents in 2017 was lower in the integration vs comparison cohorts: in the integration cohort, 296 individuals (3.7%) were long-term nursing home residents in 2017, while in the comparison cohort, 922 individuals (24.1%) were long-term nursing home residents in 2017.

**Table 1.** Characteristics of Integration and Comparison Cohorts Before and After Propensity Score Weighting

Covariates^a^	Before propensity score weighting			After propensity score weighting		
	Cohort, No. (%)^b^		Standardized mean difference^c^	Cohort, %^b^^,^^d^		Standardized mean difference^c^
	Integration (n = 7967)	Comparison (n = 3832)		Integration (n = 7967)	Comparison (n = 3832)	
Age in 2017, mean (SD), y	63.3 (14.7)	64.8 (18.6)	−0.09	63.3 (14.7)	65.8 (23.3)	0.03
Sex						
Female	5268 (66.1)	2431 (61.1)	10.5	66.1	65.5	1.1
Male	2699 (33.9)	1491 (38.9)	−10.5	33.9	34.5	−1.1
Race and ethnicity^e^						
Black	1369 (17.2)	347 (9.1)	23.1	17.2	16.8	1.1
Hispanic	22 (0.3)	50 (1.3)	−13.2	0.3	0.3	−0.1
White	6360 (79.8)	3325 (86.8)	−18.1	79.8	80.1	−0.8
Other^e^	216 (2.7)	110 (2.9)	−1.0	2.7	2.8	−0.4
Disabled^f^	5327 (66.9)	2249 (58.7)	17.0	66.9	66.1	1.5
Elixhauser Comorbidity Index in 2017, mean (SD)^g^	4.94 (3.28)	4.81 (3.59)	0.04	4.94 (3.28)	4.93 (5.15)	0.02
ADI category in 2017^h^						
Low (quintiles 1, 2, and 3 of ADI)	2990 (37.5)	1664 (43.4)	−12.1	37.5	38.6	−1.8
High (quintiles 4 and 5 of ADI)	4977 (62.4)	2168 (56.6)	12.0	62.4	61.4	1.8
Urban residence category^i^						
Rural	927 (11.6)	820 (21.4)	−27.5	11.6	11.8	−0.5
Urban	7040 (88.3)	3012 (78.6)	27.3	88.3	88.2	0.4
Nursing home care^j^: received >100 d of long-term nursing home care in 2017	296 (3.7)	922 (24.1)	−66.9	3.7	19.4	−41.7

Abbreviation: ADI, Area Deprivation Index.

^a^ Covariates used to construct propensity score weights. We weighted the comparison cohort to resemble the integration cohort on baseline covariates in 2017. See the eAppendix in Supplement 1 for details of the propensity score weighting methodology.

^b^ Number of dual-eligible individuals in the integration and comparison cohorts in 2017. eTable 1 and eFigure 3 in Supplement 1 describe the derivation and characteristics of the cohorts at each stage of exclusions.

^c^ Differences in means or proportions of each characteristic between the integration and comparison cohorts, divided by the pooled SD of the characteristic.

^d^ In the postweighted sample, characteristics for categorical variables are reported only as percentages, which allows us to appropriately display propensity score-weighted distributions of characteristics, rather than numerical frequencies, which do not reflect propensity score weighting.

^e^ Race and ethnicity assessed from administrative enrollment data.

^f^ Disability was original reason for Medicare entitlement.

^g^ Elixhauser Comorbidity Index defined as a count of 0 to 31 comorbidity indicators and characterizes chronic disease burden at baseline. Comorbidities were assessed from diagnoses on inpatient, outpatient, and professional claims from Medicare claims from UPMC (integration cohort) and the traditional Medicare program (comparison cohort) in 2017. In both cohorts, the most common comorbidities included chronic obstructive pulmonary disease, depression, diabetes, hypertension, and obesity (see eTable 2 in Supplement 1 for details).

^h^ The ADI measures socioeconomic deprivation and is constructed as a composite of demographic and economic measures. We categorized the ADI into quintiles of the national distribution of this index to identify participants in neighborhoods with a high degree of socioeconomic deprivation (highest 2 quintiles of the index) vs a lower degree of socioeconomic deprivation (bottom 3 quintiles). We linked ADI quintiles to study participants based on 9-digit zip codes reported in 2017 enrollment files.

^i^ Rurality assessed based on Rural-Urban Commuting Area codes based on the 5-digit zip codes in 2017 enrollment files.

^j^ Variable included in propensity score weighting in sensitivity analyses only.

Propensity score weighting improved the comparability of the cohorts, indicated by smaller standardized mean differences on covariates. One exception was receiving more than 100 days of nursing home care in 2017, which we did not include in propensity score weighting.

### Survival Differences Across the Cohorts

**Figure 1.**
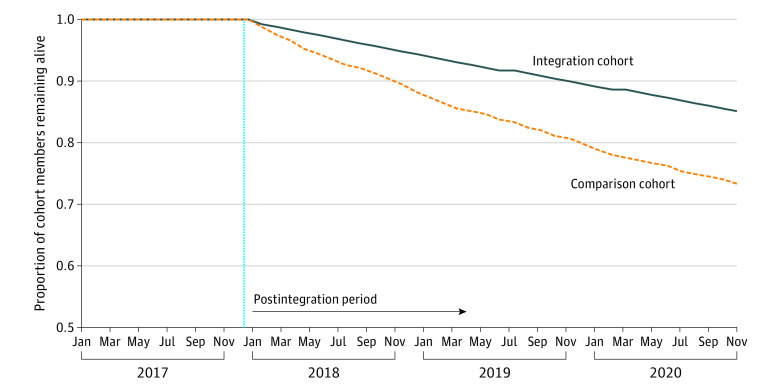
Survival Differences Between the Integration and Comparison Cohorts The figure shows the proportion of individuals in the integration vs comparison cohorts who remained alive in each study month. We used dates of death reported in administrative files to ascertain months through which members of the integration and comparison cohorts remained alive. The dashed vertical line separates the preintegration period (before 2018) from the postintegration period (2018-2020). We used a Wilcoxon rank-sum test to compare differences in survival rates between the cohorts from January 2018 to December 2020 (*P* < .001 for the difference in survival rates). Because we limited the analyses to survivors over the entire preintegration period (see Methods), survival rates during this period equal 1.

Survival rates in the cohorts diverged after January 2018 (Figure 1). By July 2018, 7720 integration cohort members (96.9%) and 3583 comparison cohort members (93.5%) remained alive—a survival difference of 3.4 percentage points. By December 2018, this difference widened to 5.3 percentage points.

### Difference-in-Differences Estimates

At baseline, individuals in the integration cohort received a mean (SD) of 2.83 (8.70) days of HCBS per month (Table 2; eTable 3 in Supplement 1). HCBS use increased differentially in the integration vs comparison cohorts from baseline through the first, second, and third postintegration years by 0.28 days per month (95% CI, 0.10-0.46; *P* = .002), 0.49 days (95% CI, 0.22-0.77; *P* < .001), and 0.61 days (95% CI, 0.28-0.94; *P* < .001), respectively. HCBS use trends were similar across the cohorts in 2017 (eFigure 4 in Supplement 1).

**Table 2.** Difference-in-Differences Estimates of Changes in Care Associated With Integrating Medicare and Medicaid Coverage

Outcome	Mean (SD) in integration cohort in 2017	Differential change from baseline^a^					
		Year 1 of integration (2018)		Year 2 of integration (2019)		Year 3 of integration (2020)	
		Estimate (95% CI)	*P* value	Estimate (95% CI)	*P* value	Estimate (95% CI)	*P* value
Receipt of home- and community-based services, d/mo^b^^,^^c^	2.83 (8.70)	0.28 (0.10 to 0.46)	.002	0.49 (0.22 to 0.77)	<.001	0.61 (0.28 to 0.94)	<.001
Care management and coordination							
Outpatient visits, No./mo^d^	0.74 (0.98)	−0.02 (−0.06 to 0.02)	.32	−0.05 (−0.11 to 0.01)	.08	−0.12 (−0.17 to −0.07)	.001
Standardized 30-d fills of chronic disease medications, No./mo^d^	3.34 (3.56)	0.01 (−0.04 to 0.07)	.59	−0.05 (−0.12 to 0.02)	.16	−0.02 (−0.10 to 0.06)	.65
Hospital visit followed within 14 d by an outpatient visit, proportion^e^	0.47 (0.48)	0.00 (−0.04 to 0.04)	.81	0.01 (−0.02 to 0.04)	.26	−0.01 (−0.04 to 0.03)	.61
Hospital and postacute care use							
Inpatient care, d/mo^b^	0.16 (1.23)	0.00 (−0.04 to 0.03)	.82	−0.02 (−0.06 to 0.03)	.45	0.03 (−0.01 to 0.07)	.06
Inpatient admissions for ambulatory care-sensitive conditions, No./mo^d^	0.01 (0.08)	0.00 (−0.01 to 0.01)	.58	0.00 (−0.01 to 0.01)	.50	0.00 (−0.01 to 0.01)	.71
Emergency department visits and observation stays, No./mo^d^	0.10 (0.39)	0.00 (−0.01 to 0.01)	.64	−0.01 (−0.02 to 0.00)	.06	−0.01 (−0.03 to 0.01)	.26
Postacute skilled nursing facility care, d/mo^b^^,^^f^	0.23 (1.93)	0.06 (−0.01 to 0.13)	.10	0.07 (0.00 to 0.13)	.05	0.03 (−0.03 to 0.08)	.34
Postacute home health care, d/mo^b^^,^^f^	1.00 (4.40)	0.14 (0.00 to 0.28)	.06	0.13 (−0.02 to 0.29)	.12	0.32 (0.15 to 0.49)	<.001
Nursing home use: long-term nursing home care, d/mo^b^^,^^c^	1.10 (5.64)	0.10 (−0.01 to 0.21)	.08	0.52 (0.25 to 0.80)	<.001	1.06 (0.74 to 1.38)	<.001

^a^ The table reports difference-in-differences estimates, which are differential changes in outcomes between the integration vs comparison cohorts from baseline through years 1, 2, and 3 of integrated coverage. The unit of analysis is the person-month. The baseline period is 2015 to 2017 for all outcomes except those measured from Medicaid claims, where the baseline period was limited to 2017 due to available data (outcomes measured using Medicaid claims are denoted by footnote c). Estimates adjusted for covariates and weighted by propensity score weights as described in the Methods and the eAppendix in Supplement 1. Robust 95% CIs account for intraperson clustering. *P* values denote the statistical significance of differential changes between the integration vs comparison cohorts from baseline through each postintegration year. See eFigure 4 in Supplement 1 for event-study plots that compare trends in outcomes between the integration vs comparison groups over the pre- and postintegration periods.

^b^ Outcome measured as the number days of service use per person-month.

^c^ Measured from Medicaid claims.

^d^ Outcome measured as a count per person-month (for example, number of outpatient visits per person-month).

^e^ Proportion with an outpatient visit within 14 days of a hospital visit. Hospital visits included inpatient admissions, emergency department visits, or observation stays. The denominator of this measure is index hospital visits per person-month. Index hospital visits in December 2020 were excluded because outcomes after December 31, 2020, are censored.

^f^ Excludes postacute care services in December 2020 because end dates for services completed after December 31, 2020, are censored.

At baseline, individuals in the integration cohort had a mean (SD) of 0.74 (0.98) outpatient visits per month, filled a mean (SD) of 3.34 (3.56) medications for chronic conditions per month, and the mean (SD) proportion of patients who had a follow-up outpatient visit after a hospital stay was 0.47 (0.48) (Table 2). We did not observe differential changes in medication use or follow-up outpatient visits after hospitalization between the cohorts through the first, second, or third postintegration years, while outpatient visits declined differentially in the integration vs comparison cohorts after 3 years.

Hospital utilization and postacute care in skilled nursing facilities did not change differentially across the cohorts. In the integration cohort, there was a differential increase in postacute home health use by the third postyear (2020), coinciding with the COVID-19 pandemic. However, it is unclear whether this reflects a change associated with integration vs a pandemic-associated shift in postacute care toward home-based settings.^34^

Finally, there was a differential increase in days of long-stay nursing home care in the integration cohort, reflecting a greater decline in days in the comparison vs integration cohorts after 2017. However, nursing home residents experienced high mortality, particularly in the comparison cohort (eFigure 5 in Supplement 1).

### Sensitivity of Estimates to Differential Attrition From Mortality

**Figure 2.**
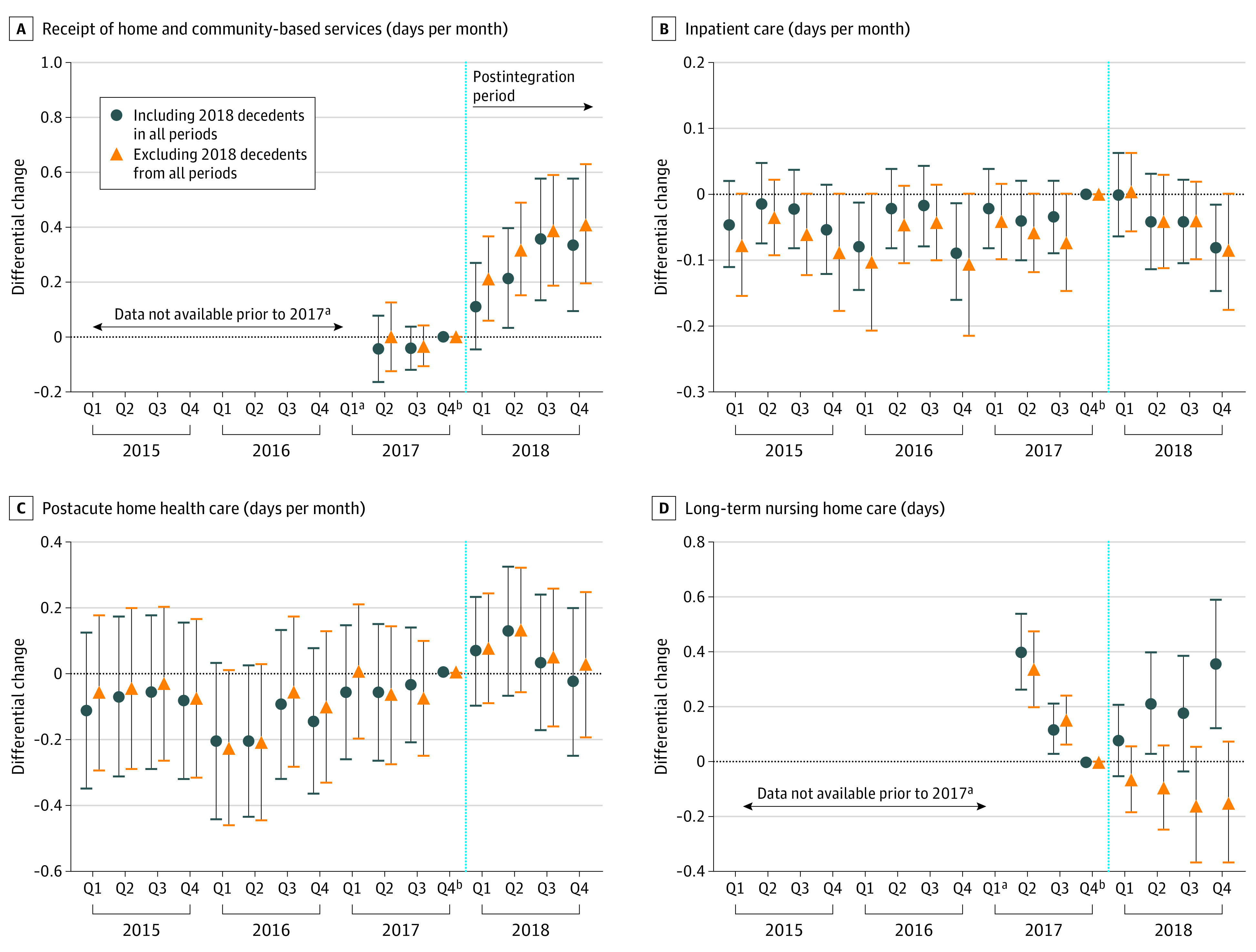
Comparison of Differential Changes in Outcomes From 2015 to2018 Before vs After Excluding Decedents in 2018 From All Periods Each graph displays the adjusted differential change in the outcome by quarter in the integration vs comparison cohorts, relative to the first quarter of 2017 (the reference period). Estimates were obtained from event-study models, which were adjusted for covariates and weighted by propensity score weights. Error bars represent 95% CIs for the estimates of differential changes. Dots denote estimates from our main analysis sample, which included decedents in the postintegration period. Triangles denote estimates from a sensitivity analysis sample, which excluded decedents in 2018 from all periods. Dashed vertical lines separate the preintegration period (2015-2017) from the postintegration period (2018). See eFigure 6 in Supplement 1 for plots of all study outcomes. ^a^The baseline period was 2017 for days of home- and community-based services and days of long-term nursing home care (both measured in Medicaid claims) due to available data. For other outcomes, the baseline period was 2015 to 2017. ^b^Each graph displays the adjusted differential change in the outcome by quarter (Q) in the integration vs comparison cohorts, relative to the first quarter of 2017 (the reference period).

Figure 2 explores whether estimates were sensitive to differential attrition from mortality, focusing on the first postintegration year when any plan effects on mortality likely were minimal. We found similar differential increases in HCBS use when we included vs excluded individuals who died in 2018 from all periods. However, we no longer saw a differential increase in nursing home days when we excluded individuals who died in 2018. Plots for other outcomes are in eFigure 6 in Supplement 1.

### Supplementary Analyses

In analyses limited to community-dwelling dual-eligible individuals at baseline, we continued to observe a differential increase in HCBS use, and no differential changes in medication use for chronic conditions, follow-up outpatient care after a hospital stay, or hospital use (Table 3). In the integration cohort, increases in HCBS use were greatest among individuals who lived in the community and enrolled in an HCBS waiver program in 2017 (eTable 5 in Supplement 1).

**Table 3.** Sensitivity Analysis Among Dual-Eligible Individuals Living in the Community at Baseline (2017)^a^

Outcome	Mean (SD) in integration cohort in 2017	Differential change from baseline^b^					
		Year 1 of integration (2018)		Year 2 of integration (2019)		Year 3 of integration (2020)	
		Estimate (95% CI)	*P* value	Estimate (95% CI)	*P* value	Estimate (95% CI)	*P* value
Receipt of home- and community-based services, d/mo	2.92 (8.81)	0.31 (0.12 to 0.50)	.001	0.56 (0.25 to 0.87)	<.001	0.77 (0.38 to 1.17)	<.001
Care management and coordination							
Outpatient visits, No./mo	0.76 (0.99)	−0.03 (−0.06 to 0.00)	.05	−0.04 (−0.07 to −0.01)	.02	−0.09 (−0.13 to −0.05)	.001
Standardized 30-d fills of chronic disease medications, No./mo	3.29 (3.58)	0.03 (−0.03 to 0.09)	.26	0.00 (−0.07 to 0.08)	.97	0.01 (−0.07 to 0.09)	.81
Hospital visit followed within 14 d by an outpatient visit, proportion	0.48 (0.49)	−0.01 (−0.04 to 0.02)	.53	0.02 (−0.02 to 0.06)	.29	0.00 (−0.04 to 0.03)	.88
Hospital and postacute care use							
Inpatient care, d/mo	0.15 (1.14)	−0.04 (−0.09 to 0.00)	.07	−0.03 (−0.08 to 0.02)	.21	0.03 (−0.01 to 0.07)	.13
Inpatient admissions for ambulatory care–sensitive conditions, No./mo	0.005 (0.07)	0.00 (−0.01 to 0.01)	.56	0.00 (−0.01 to 0.01)	.88	0.00 (−0.01 to 0.01)	.52
Emergency department visits and observation stays, No./mo	0.10 (0.39)	0.00 (−0.01 to 0.01)	.68	−0.01 (−0.02 to 0.00)	.07	−0.01 (−0.02 to 0.00)	.05
Postacute skilled nursing facility care, d/mo	0.18 (1.64)	0.02 (−0.04 to 0.08)	.47	0.00 (−0.06 to 0.05)	.89	−0.01 (−0.07 to 0.04)	.62
Postacute home health care, d/mo	0.78 (3.87)	0.04 (−0.15 to 0.23)	.68	0.08 (−0.12 to 0.29)	.43	0.42 (0.21 to 0.63)	<.001
Nursing home use: long-term nursing home care, d/mo^c^	0.05 (1.15)	−0.01 (−0.10 to 0.08)	.81	−0.10 (−0.29 to 0.09)	.30	−0.18 (−0.50 to 0.13)	.24

^a^ The table reports estimates from a sensitivity analysis in which the sample was limited to dual-eligible individuals who lived in the community in 2017 (n = 7639 individuals in the integration cohort and n = 2847 individuals in the comparison cohort). Dual-eligible individuals who lived in a nursing home in 2017 were excluded. See the notes to Table 2 for details about the outcomes examined.

^b^ Difference-in-differences estimates are differential changes in outcomes between the integration vs comparison cohorts from baseline through years 1, 2, and 3 of integrated coverage. Estimates adjusted for covariates and weighted by propensity score weights. Robust 95% CIs account for intraperson clustering. eTable 4 in Supplement 1 shows baseline characteristics of the community-dwelling integration and comparison cohorts before and after propensity score weighting.

^c^ Difference-in-differences estimates for the overall sample (Table 2) are a weighted average of estimates from the subsamples of community-dwelling and nursing facility–dwelling individuals who remained alive in each study period (eTable 5 in Supplement 1). Because of mortality differences across the subsamples during the postintegration period, the weight assigned to individuals who initially lived in nursing homes decreased over time (eFigure 5 in Supplement 1). The smaller representation of nursing home residents over time, particularly in the comparison cohort where mortality was highest, explains the differential increase in long-term nursing home use in the overall sample (Table 2), whereas difference-in-differences estimates for the separate community-dwelling and nursing facility subsamples were small and statistically insignificant (eTable 5 in Supplement 1).

Among community-dwelling individuals in an HCBS waiver program in 2017, the proportion of those classified as HCBS-eligible by 2020 was 3.0% higher in the integration vs comparison cohorts (eFigure 7 in Supplement 1). Among community-dwelling individuals enrolled in HCBS programs in 2017, the proportion who became eligible for long-term nursing home care by 2020 was 3.9% lower, and the proportion who remained eligible for and receiving HCBS by 2020 was 4.3% higher, in the integration vs comparison cohorts. Results were similar when we limited analyses to survivors over the 2018 to 2020 period.

## Discussion

This cohort study using a difference-in-differences analysis found that the integration of Medicare and Medicaid coverage for dual-eligible individuals was associated with differential increases in use of HCBS after 3 years, relative to changes in a comparison cohort of dual-eligible individuals maintaining nonintegrated coverage. However, we did not see differential reductions in hospitalizations or improvements in care management and coordination, including medication use for chronic conditions or follow-up care after a hospital stay, in the integration vs comparison cohorts. Differences in mortality across the cohorts, likely attributable to unmeasured risk factors that preceded integration, limited our ability to determine whether integration was associated with changes in long-term nursing home stays. Altogether, these results demonstrate some benefits of integration, particularly in facilitating increased engagement with community-based services and supports, but they also highlight opportunities to improve how integrated programs manage care and a need to further evaluate their performance.

In addition to these primary findings, our descriptive analyses of long-term care eligibility data revealed that higher proportions of integration vs comparison cohort members remained or became eligible for and received HCBS from 2018 to 2020. Further, among individuals enrolled in HCBS programs at baseline, smaller proportions of those in the integration vs comparison cohorts eventually qualified for long-term nursing home care. These findings are notable because both cohorts were in the same Medicaid managed care plan from 2018 to 2020. Thus, differences in long-term care eligibility may reflect the influence of integration. For example, having 1 plan manage Medicare and Medicaid services for the same people could have enhanced the plan’s capacity to monitor changes in health, initiate referrals for HCBS eligibility, and extend the period over which dual-eligible individuals received long-term care in the community before needing nursing home care.

Our results are consistent with prior studies showing an association of integrated programs with increased HCBS use.^9,17,18^ These patterns are conceptually consistent with integrated plans’ incentives to manage long-term care in less-resource-intensive settings (eg, the community rather than nursing homes)^6,14,15,16^ and may also be a positive sign for patients, who often prefer to receive long-term care in the community.^35,36^ Our findings are also consistent with studies that reported mixed or null changes in changes in hospitalizations and care coordination associated in other integrated models.^18,19,20,21^ Although it may take time for changes in care management to have a measurable effect on hospitalizations, our study did not find improvements on leading indicators of care coordination, such as follow-up outpatient care after a hospital stay. Therefore, our findings and prior evidence underscore an opportunity for integrated programs to strengthen care coordination and management of hospital utilization.

This study identified differences between individuals who joined UPMC’s integrated plan vs those in the comparison cohort (traditional Medicare), including statistically significantly lower long-term nursing home use in the integration vs comparison cohorts at baseline. Such differences in nursing home use may be associated with health, functional, and cognitive factors and suggest that enrollees in UPMC’s D-SNP were healthier than enrollees in traditional Medicare at baseline. These patterns are consistent with national analyses that found favorable selection in Medicare Advantage (ie, better health among enrollees in Medicare Advantage than in traditional Medicare).^37^ Although our difference-in-differences approach controls for cohort differences that remain constant over time, unmeasured factors that are correlated with mortality and utilization trends could bias our estimates. This was a particular concern when we examined long-term nursing home stays, for which our analyses were sensitive to mortality differences that likely reflected health factors present at baseline. Research leveraging random or quasirandom assignment to integrated programs (for example, variation in default enrollment) could help to control for these unmeasured differences and provide more robust evidence on integrated program performance.^16^

### Limitations

Our study had several limitations. First, unmeasured baseline factors associated with outcome trends, and time-varying factors differing across the integration and comparison cohorts, could have biased our findings. Second, the final year of our analysis coincided with the start of the COVID-19 pandemic. Changes in care during this year, including shifts in postacute care from skilled nursing facilities to home-based settings, might have differentially influenced care patterns across the cohorts, obscuring changes associated with integration. Third, our study was limited to a FIDE-SNP in 1 state operated by a health insurer affiliated with a large health system. Unique features of this plan and setting may not generalize to other FIDE-SNPs. However, the FIDE-SNP we examined was the largest in the US, making it an important case study of an integrated plan.

## Conclusions

This cohort study showed that after 3 years, dual-eligible individuals in an integrated plan in Pennsylvania had greater increases in HCBS vs a comparison cohort maintaining nonintegrated coverage. However, integration was not associated with changes in hospitalizations or care management, and we could not ascertain changes in long-term nursing home stays attributable to integration. Our findings demonstrate some benefits of integrated coverage but also highlight opportunities to strengthen how these models manage care and a need to further evaluate the performance of these models.

## Notes

Supplement 1. **eAppendix. **eMethods and eResults **eFigure 1.** Policy context and timeline **eFigure 2.** Data file linkages **eFigure 3.** Study inclusion flowsheet **eTable 1.** Sample characteristics after exclusion steps **eTable 2.** Most common comorbidities in integration and comparison cohorts, 2017 **eTable 3.** Difference-in-differences estimates of changes in care associated with integrating Medicare and Medicaid coverage **eFigure 4.** Estimates from event-study models **eFigure 5.** Survival plots stratified according whether dual eligible individuals in 2017 resided in a nursing home, lived in the community and were enrolled in an HCBS waiver program, or lived in the community and were not in an HCBS waiver program **eFigure 6.** Comparison of adjusted trends in outcomes in the integration and comparison cohorts, 2015-2018, before and after excluding decedents in 2018 **eTable 4.** Characteristics of sensitivity analysis sample: dual eligible individuals living in the community at baseline (2017) **eTable 5.** Difference-in-differences estimates, stratified according whether dual eligible individuals in 2017 resided in a nursing home, lived in community and were enrolled in an HCBS waiver program, or lived in community and not in an HCBS waiver program **eFigure 7.** Monthly changes in long-term care eligibility among dual eligible individuals in the integration and comparison cohorts, 2018-2020
eReferences.

Supplement 2.
Data Sharing Statement
