# Supplementary material for: Changes in Care Associated With Integrating Medicare and Medicaid for Dual-Eligible Individuals
Source: JAMA Health Forum. 2023 Dec 21;4(12):e234583. doi: 10.1001/jamahealthforum.2023.4583 (PMC10739174; doi:10.1001/jamahealthforum.2023.4583)
Supplement: Supplement 1. — eAppendix. eMethods and eResults eFigure 1. Policy context and timeline eFigure 2. Data file linkages eFigure 3. Study inclusion flowsheet eTable 1. Sample characteristics after exclusion steps eTable 2. Most common comorbidities in integration and comparison cohorts, 2017 eTable 3. Difference-in-differences estimates of changes in care associated with integrating Medicare and Medicaid coverage eFigure 4. Estimates from event-study models eFigure 5. Survival plots stratified according whether dual eligible individuals in 2017 resided in a nursing home, lived in the community and were enrolled in an HCBS waiver program, or lived in the community and were not in an HCBS waiver program eFigure 6. Comparison of adjusted trends in outcomes in the integration and comparison cohorts, 2015-2018, before and after excluding decedents in 2018 eTable 4. Characteristics of sensitivity analysis sample: dual eligible individuals living in the community at baseline (2017) eTable 5. Difference-in-differences estimates, stratified according whether dual eligible individuals in 2017 resided in a nursing home, lived in community and were enrolled in an HCBS waiver program, or lived in community and not in an HCBS waiver program eFigure 7. Monthly changes in long-term care eligibility among dual eligible individuals in the integration and comparison cohorts, 2018-2020 eReferences. [file jamahealthforum-e234583-s001.pdf]

Supplemental Online Content

Roberts ET, Xue L, Lovelace J, et al. Changes in care associated with integrating Medicare and Medicaid for dual-eligible individuals. *JAMA Health Forum.* Published online December 21, 2023. doi:10.1001/jamahealthforum.2023.4583

eAppendix. eMethods and eResults

eFigure 1. Policy context and timeline

eFigure 2. Data file linkages

eFigure 3. Study inclusion flowsheet

eTable 1. Sample characteristics after exclusion steps

eTable 2. Most common comorbidities in integration and comparison cohorts, 2017

eTable 3. Difference-in-differences estimates of changes in care associated with integrating Medicare and Medicaid coverage

eFigure 4. Estimates from event-study models

eFigure 5. Survival plots stratified according whether dual eligible individuals in 2017 resided in a nursing home, lived in the community and were enrolled in an HCBS waiver program, or lived in the community and were not in an HCBS waiver program

eFigure 6. Comparison of adjusted trends in outcomes in the integration and comparison cohorts, 2015-2018, before and after excluding decedents in 2018

eTable 4. Characteristics of sensitivity analysis sample: dual eligible individuals living in the community at baseline (2017)

eTable 5. Difference-in-differences estimates, stratified according whether dual eligible individuals in 2017 resided in a nursing home, lived in community and were enrolled in an HCBS waiver program, or lived in community and not in an HCBS waiver program

eFigure 7. Monthly changes in long-term care eligibility among dual eligible individuals in the integration and comparison cohorts, 2018-2020

eReferences.

This supplemental material has been provided by the authors to give readers additional information about their work.

© 2023 Roberts ET et al. *JAMA Health Forum.*

I. Study Setting and Policy Context

We studied a FIDE-SNP operated by UPMC *For You* (hereafter, UPMC), an insurer that operates Medicare and Medicaid managed care plans in Pennsylvania. UPMC's FIDE-SNP operates under Medicare Advantage contract H4279. UPMC has operated a D-SNP under this contract since 2013 (eFigure 1).[a] By 2015—the start of our study period—this D-SNP enrolled approximately 18,000 dual eligible individuals, largely in southwestern Pennsylvania.[1] At the contract's inception, this D-SNP covered Medicare benefits only.

The policy change motivating our analysis occurred in 2018, when Pennsylvania introduced Community HealthChoices (CHC), a comprehensive Medicaid managed care program that included coverage of long-term services and supports (LTSS). CHC was designed to manage the financing and delivery of LTSS and physical health services for older adults and disabled individuals—most of whom are dually eligible for Medicare and Medicaid—under a managed care model. The primary goals of CHC were to expand the provision of LTSS in community-based settings (e.g., via home- and community-based services [HCBS]); to enhance the coordination of LTSS and physical health services; and to promote efficient care delivery in Medicaid.[2,3] Behavioral health care is managed separately from CHC.

CHC replaced a fee-for-service Medicaid program in Pennsylvania, under which there had been little opportunity to integrate Medicaid financing and benefits with Medicare. (Under the fee-for-service Medicaid program, individuals could receive HCBS through one of several waiver programs, in which enrollment was capped. CHC eliminated these HCBS waivers and caps on enrollment, and moved coverage of these services under managed care plans.) In the CHC program, three managed care insurers, including UPMC, operated statewide Medicaid

[a] UPMC has operated D-SNPs since 2006, but the plan contract we studied was a D-SNP since 2006 and our integration cohort remained enrolled in this D-SNP continuously during our baseline study period..

© 2023 Roberts ET et al. *JAMA Health Forum.*

managed care plans. Medicaid beneficiaries could choose among these CHC plans; beneficiaries were assigned a plan administratively if they did not choose one.[2] Insurers operating CHC plans were also required to offer companion D-SNPs. Although individuals were not required to enroll in a D-SNP affiliated with their CHC plan, CHC included provisions to promote integrated care, such as assigning individuals to the same primary care providers in CHC and D-SNPs (when enrolled in D-SNPs and CHC plans operated by the same parent insurer).[4] Thus, CHC increased opportunities to integrate coverage for dual eligible individuals in Pennsylvania.[5]

Community HealthChoices was introduced in phases, beginning in January 2018 in 14 counties in southwestern Pennsylvania,[2] where UPMC's D-SNP operated since 2013. In 2018, approximately 85% of existing enrollees in this D-SNP chose or were administratively assigned to UPMC's Medicaid managed care plan. (Because the state managed the enrollment process, we cannot observe administrative plan assignments in our Medicaid data, which we obtained from UPMC.) Of approximately 11,000 full-benefit dual eligible individuals who 1) lived in the 14-county region of Pennsylvania where CHC was initially introduced and 2) had continuous enrollment in UPMC's D-SNP from 2015-2017, approximately 9,300 joined UPMC's CHC plan on January 1, 2018. Our *integration cohort*, for whom UPMC managed both Medicare spending and Medicaid spending starting in 2018, is drawn from the population of established D-SNP enrollees who joined UPMC's CHC plan in 2018 (see Section III for details).

At the time of CHC's introduction, UPMC applied to CMS to be classified as a FIDE-SNP, a designation given to D-SNPs that manage Medicaid-covered spending when either the D-SNP or its parent insurer has a capitation contract with a state Medicaid program.[6] UPMC's FIDE-SNP classification was finalized by CMS in 2020. However, because UPMC managed Medicare and Medicaid spending for individuals in the integration cohort since 2018, we

© 2023 Roberts ET et al. *JAMA Health Forum.*

analyzed the entire 2018-2020 period as the post-integration period for this cohort. In 2023, UPMC's FIDE-SNP (contract H4279) served approximately 39,000 dual eligible individuals throughout Pennsylvania.[7]

FIDE-SNPs are statutorily eligible to receive a Medicare frailty payment adjustment if they serve individuals with similar or greater levels of frailty than Programs of All-Inclusive Care of the Elderly (PACE). D-SNPs that do not meet FIDE designation are ineligible for this frailty payment adjustment.[8] However, not all FIDE-SNPs receive this adjustment, and UPMC's FIDE-SNP did not receive it during our study period or during 2021-2023. Thus, there was no substantive change in Medicare payments to the UPMC plan between 2018-19 (while its FIDE-SNP designation was pending) to 2020 (after this designation was finalized). The substantive policy change in our study was the 2018 reform that mandated Medicaid managed care enrollment and enabled UPMC to insure dual eligible individuals through companion Medicare and Medicaid managed care plans.

© 2023 Roberts ET et al. *JAMA Health Forum.*

eFigure 1: Policy context and timeline

Year	2013	…	2015	…	2017	2018	…	2020
**Milestone/ Notes**	▪ UPMC D-SNP established (primarily serves southwestern PA)		▪ UPMC D-SNP serves ~18,000 dual eligibles<br>▪ Start of baseline period		▪ UPMC D-SNP serves ~22,000 dual eligibles<br>▪ ~11,000 full-benefit dual eligibles in SW PA continuously enrolled in UPMC D-SNP from 2015-17 (pop'n [A])<br>▪ End of baseline period	▪ PA starts Community HealthChoices (CHC), a Medicaid managed care program that included LTSS coverage<br>▪ CHC run by 3 statewide insurers, including UPMC<br>▪ CHC insurers also required to operate D-SNPs<br>▪ From pop'n [A], 9,300 dual eligibles join UPMC's companion CHC plan 1/2018<br>▪ Start of post-integration period		▪ UPMC D-SNP formally designated as a FIDE-SNP<br>▪ Total UPMC FIDE-SNP enrollment (statewide): ~30,000 dual eligibles<br>▪ Enrollment grows to 39,000 dual eligibles statewide by 2023
			← *Baseline period* →			← *Post-integration period* →		

Note: SW PA=southwestern Pennsylvania.

© 2023 Roberts ET et al. *JAMA Health Forum.*

II. Data

We analyzed Medicare and Medicaid administrative data from four main sources. First, we obtained Medicare enrollment and claims from UPMC's D-SNP (2015-2019) and FIDE-SNP (2020). Second, we obtained 2018-2020 Medicaid enrollment and claims for UPMC's CHC Medicaid managed care plan. Third, to measure baseline service use in Medicaid, we analyzed a Medicaid service history file, which reported 12 months of claims history from Pennsylvania's fee-for-service Medicaid program (predecessor to CHC) for individuals who joined UPMC's CHC Medicaid managed care plan in 2018.[b] This file also reported Medicaid HCBS waiver program eligibility and nursing home residence in 2017. Fourth, we obtained 2015-2020 Medicare enrollment and fee-for-service claims for our comparison cohort of traditional Medicare enrollees.

We linked these data using an encrypted person-level identifier. To identify administrative enrollment and claims files for dual eligible individuals with traditional Medicare, the encrypted identifier was keyed to individual-level Social Security Numbers and sent as an encrypted finder file to General Dynamics Information Technology, CMS' data contractor. After extracting records for dual eligible individuals with traditional Medicare, the contractor removed Social Security Numbers, retaining only an encrypted person identifier in files sent to our study team. This enabled us to link enrollment and claims files for traditional Medicare to UPMC data. eFigure 2 illustrates linkages of our data sources.

[b] The service history file reported 12 months of claims history, but we found it to be incomplete for the first 3 months of 2017, particularly for long-term nursing home stays. Our conversations with UPMC suggested that this was likely because index stays beginning prior to January 2017 and continuing into 2017 were not consistently included in the file. Therefore, for all analyses of Medicaid service utilization, we allowed a 90-day run-in period, meaning that analyses of these services began in the second quarter of 2017.

© 2023 Roberts ET et al. *JAMA Health Forum.*

eFigure 2: Data file linkages

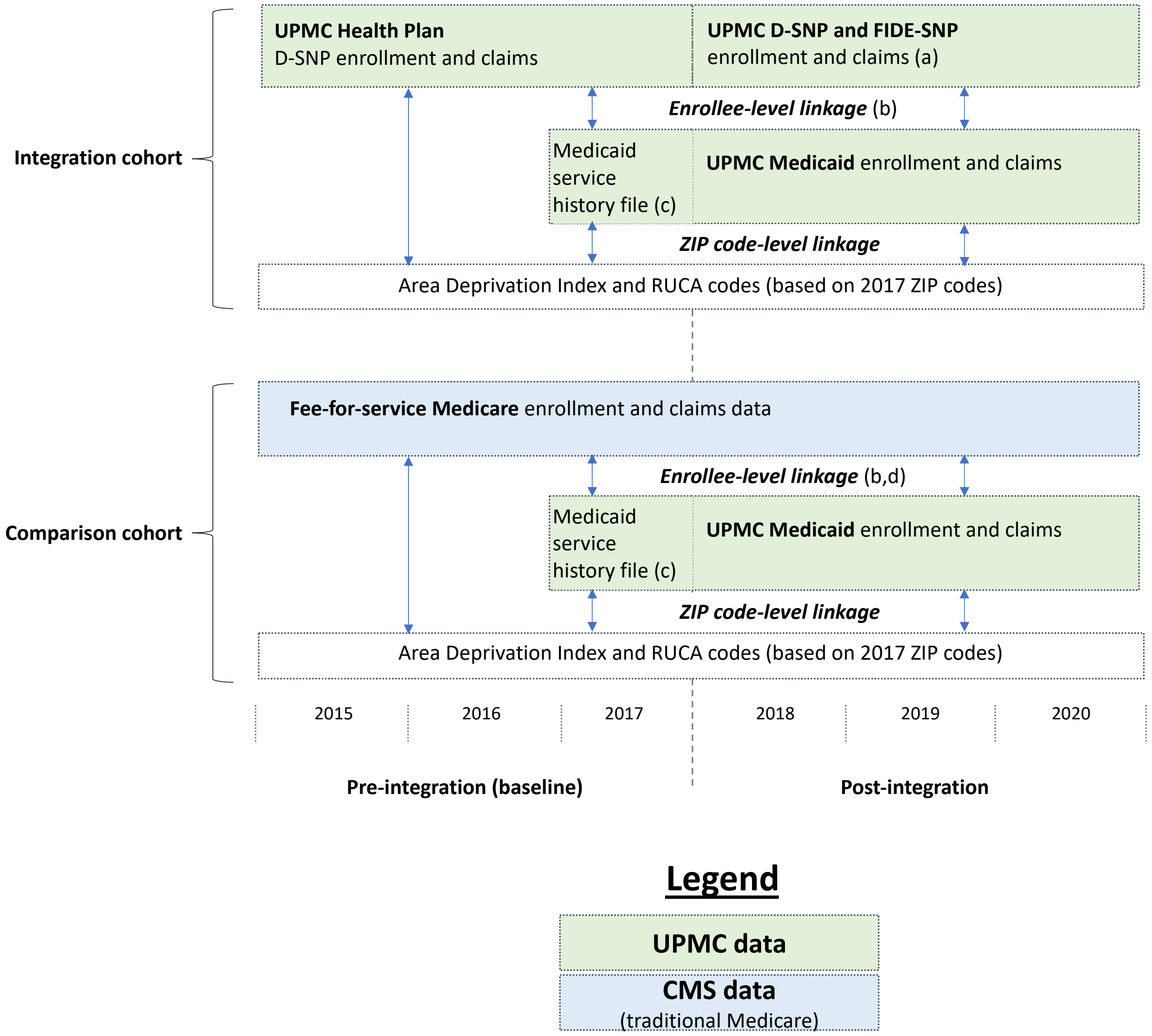

[a] UPMC's D-SNP was formally designated as a FIDE-SNP in 2020. However, because UPMC managed Medicare and Medicaid spending for dual eligible individuals via companion managed care plans starting in 2018, we analyzed 2018-2020 as a post-integration period.
[b] Enrollee-level linkages conducted using an encrypted person-level identifier.
[c] Medicaid service history files reported 12 months of claims history from Pennsylvania's prior fee-for-service Medicaid program. The files also reported Medicaid HCBS waiver eligibility and nursing home residence in 2017. The files were originally created by the Pennsylvania Medicaid program and provided to managed care insurers preparing to establish Medicaid managed care plans under Pennsylvania's Community HealthChoices program.
[d] To identify records for enrollees in traditional (i.e., fee-for-service Medicare), UPMC provided CMS with file of Social Security Numbers for enrollees in its Medicaid managed care plan, which was keyed to the encrypted person-level identifier. A CMS data contractor extracted records matching these Social Security Numbers and returned files to us containing only the encrypted person-level identifier, which we then used to link fee-for-service Medicare claims to UPMC Medicaid enrollment and claims and the Medicaid service history file.

© 2023 Roberts ET et al. *JAMA Health Forum.*

We also linked administrative files to the Area Deprivation Index (ADI)[9] and Rural-Urban Commuting Area (RUCA) codes from the US Department of Agriculture to measure community-level socioeconomic deprivation and rurality, respectively.[10] The ADI was linked at the 9-digit ZIP code level and RUCA codes were linked at the 5-digit ZIP code level based on person-level residence information reported in 2017 enrollment files.

III. Inclusion criteria

We analyzed two cohorts, which were defined as follows:

1) The *integration cohort* included dual eligible individuals enrolled in UPMC's D-SNP in the baseline period (2015-2017) who were continuously enrolled, while alive, in UPMC's D-SNP and Medicaid managed care plan in the post-integration period (2018-2020).
2) The *comparison cohort* included dual eligible individuals with traditional Medicare at baseline who were continuously enrolled, while alive, in traditional Medicare and UPMC's Medicaid managed care plan in the post-period.

Limiting analyses to enrollees in the D-SNP or traditional Medicare at baseline, and who maintained consistent D-SNP or traditional Medicare coverage (while alive) through 2020, avoids comparing dual eligible individuals who might have switched Medicare coverage once a companion Medicaid plan became available (these plan switches may be endogenous to outcomes). **Both cohorts were limited to dual eligibles enrolled in full Medicaid in all study months while alive.**

eFigure 3 shows the derivation of our sample and eTable 1 compares characteristics of dual eligible individuals retained in the sample at each stage of exclusions.

© 2023 Roberts ET et al. *JAMA Health Forum.*

eFigure 3: Study inclusion flowsheet

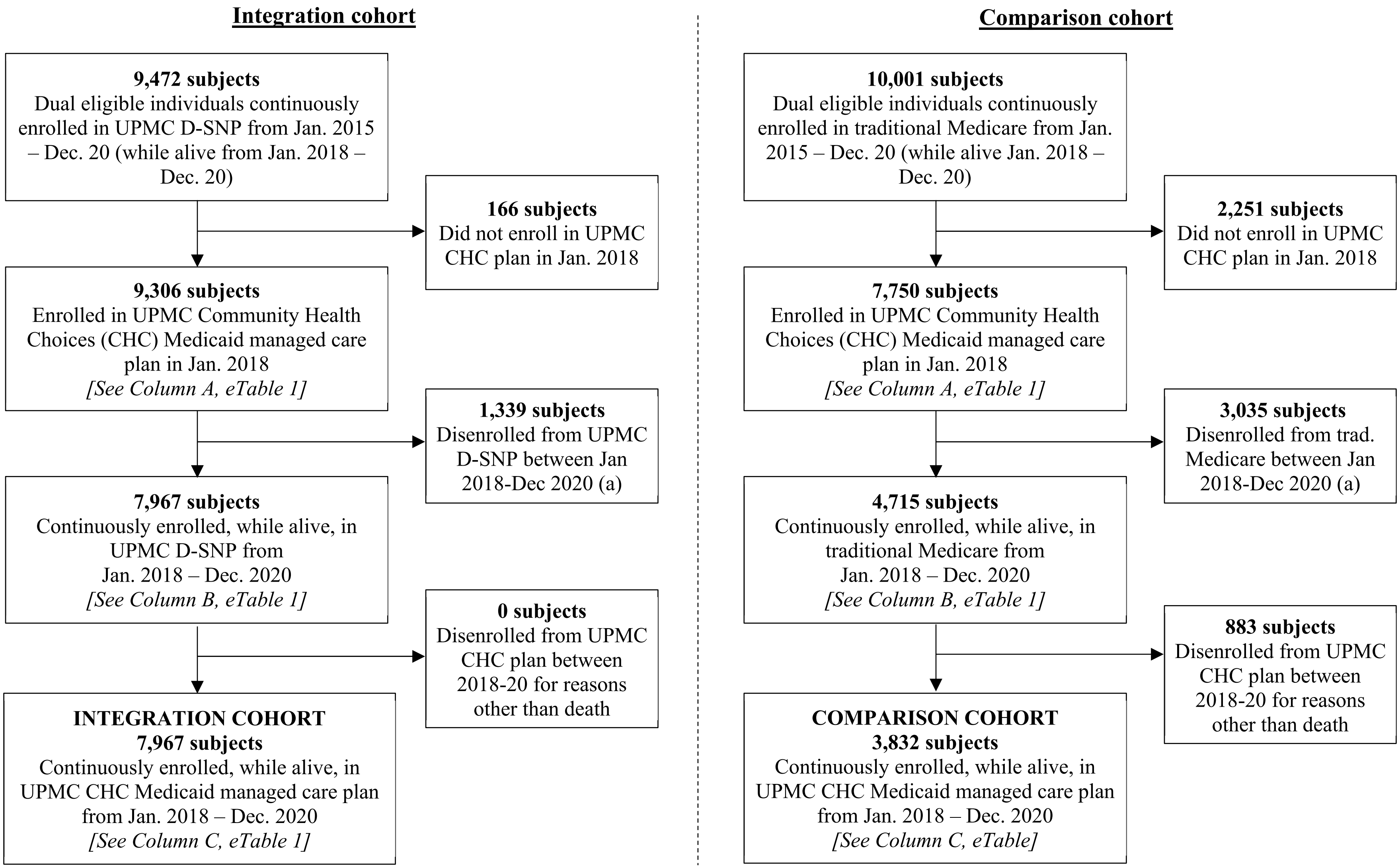

eTable 1 displays characteristics of the integration and comparison cohorts at each stage of our exclusion criteria to assess the implications of exclusions on sample representativeness. (a) That is, a study subject disenrolled from fee-for-service Medicare or a UPMC plan between January 2018 and December 2020 (for decedents, this means disenrollment prior to death).

© 2023 Roberts ET et al. *JAMA Health Forum.*

eTable 1: Sample characteristics after exclusion steps

	Column (reflects stage of exclusions in eFigure 3)					
	(A)		(B)		(C)	
	Integration Cohort (n=9,306)	Comparison Cohort (n=7,750)	Integration Cohort (n=7,967)	Comparison Cohort (n=4,715)	FINAL Integration Cohort (n=7,967)	FINAL Comparison Cohort (n=3,832)
**Covariates:**						
Mean age in 2017 (years)	63.3	61.3	63.3	63.3	63.3	64.8
Female sex, %	66.1%	59.2%	66.1%	59.3%	66.1%	61.1%
Male sex, %	33.9%	40.8%	33.9%	40.7%	33.9%	38.9%
Race/ethnicity, % category:						
Non-Hispanic Black	17.2%	10.1%	17.2%	9.8%	17.2%	9.1%
Hispanic	0.3%	1.1%	0.3%	1.3%	0.3%	1.3%
Non-Hispanic White	79.8%	85.7%	79.8%	86.0%	79.8%	86.8%
Other	2.7%	3.0%	2.7%	2.9%	2.7%	2.9%
Disabled, %	66.9%	66.6%	66.9%	61.8%	66.9%	58.7%
Elixhauser Comorbidity Index in 2017	4.94	4.50	4.94	4.69	4.94	4.81
Area Deprivation Index (ADI) in 2017, % category:						
Low (quintiles 1, 2, and 3 of ADI)	37.5%	42.3%	37.5%	43.4%	37.5%	43.4%
High (quintiles 4 and 5 of ADI)	62.4%	57.7%	62.4%	56.6%	62.4%	56.6%
Urban residence, % in category:						
Rural	11.6%	21.7%	11.6%	21.3%	11.6%	21.4%
Urban	88.3%	78.3%	88.3%	78.7%	88.3%	78.6%
**Long-term nursing home care:**						
More than 100 days of long-term nursing home care in 2017, %	3.7%	15.3%	3.7%	21.4%	3.7%	24.1%

See the notes to Table 1 in the main manuscript for variable definitions. Sample characteristics are measured for individuals in 2017 and shown without propensity score weighting.

© 2023 Roberts ET et al. *JAMA Health Forum.*

eTable 2: Most common comorbidities in integration and comparison cohorts, 2017

Integration Cohort		Comparison cohort	
**Comorbidity**	**Prevalence in 2017 (%)**	**Comorbidity**	**Prevalence in 2017 (%)**
**Hypertension**	67%	**Hypertension**	63%
**Obesity**	47%	**Other neurological disorders**	34%
**Chronic pulmonary disease**	41%	**Depression**	34%
**Depression**	35%	**Deficiency Anemias**	32%
**Diabetes w/o chronic complications**	32%	**Obesity**	31%
**Deficiency Anemias**	26%	**Peripheral vascular disease**	30%
**Hypothyroidism**	24%	**Diabetes w/o chronic complications**	29%
**Diabetes w/ chronic complications**	24%	**Chronic pulmonary disease**	29%
**Other neurological disorders**	23%	**Hypothyroidism**	24%
**Psychoses**	23%	**Fluid and electrolyte disorders**	21%

Prevalence of comorbidities from a set of 31 comorbidities that comprise the Elixhauser Comorbidity Index. The Elixhauser Comorbidity index is a method of characterizing patients' chronic disease burden based on diagnoses coded in administrative data. Comorbidities were assessed from diagnoses on inpatient, outpatient, and professional claims from UPMC D-SNP (integration cohort) and fee-for-service Medicare (comparison cohort) in 2017. Here, we report the frequencies of the 10 most common Elixhauser comorbidities in the integration and comparison cohorts. Prevalence in 2017 represents the proportions of integration and comparison cohort members with a diagnosis for the condition in 2017.

© 2023 Roberts ET et al. *JAMA Health Forum.*

IV. Variables

This section defines our outcome variables and covariates.

Outcome variables

We examined 10 outcomes, each of which was measured at the person-month level. The denominator for all outcomes included person-months in which dual eligible individuals were alive and met the study enrollment criteria described in Section III. Accompanying the definition of each outcome measure, we provided citations to representative evaluations of integrated care programs that analyzed this measure, or a very closely related measure.

1. **Receipt of Medicaid-covered home and community-based services (HCBS)[11,12] (days)**
   - We counted the number of unique days per person-month in which individuals received any Medicaid-covered HCBS during the period 2017-2020
     - 2018-2020 outcomes measured from UPMC Medicaid managed care files
     - 2017 is baseline, measured from Medicaid service history file (for Pennsylvania's predecessor fee-for-service Medicaid program)
   - Because of the complexity of how HCBS is billed, we counted the number of days of any service use, per Wysocki et al.[13]
   - Measured from UPMC Member Medical claims /Medicaid service history file where:
     - Procedure code on claim matches to one of the codes in the table below
     - In UPMC Member Medical claims, restrict to records where line of business (CLAIM_LOB) = CHC or Medicaid (MA)

HCBS category	CPT Procedure Codes
Personal assistance services	W1792 and W1793
Adult Daily Living	S5102
Home health	T1002, T1003, and T2025
Residential habilitation	W0100, W0101, W0102, W0103, W0104, W0105

Note: Personal assistance services accounted for >98% of all HCBS services in our population. Residential habilitation may include Medicaid-covered physical therapy and occupational therapy.

2. **Outpatient visits [11,12,14-17] (count)**
   - We counted the number of person-days per month in which individuals had an outpatient evaluation and management visits in a physician's office, hospital outpatient department, Federally Qualified Health Center (FQHC), or rural health

clinic. (We counted days per person-month to avoid counting potentially duplicate claims billed on the same day.)

- Measured from UPMC D-SNP/FIDE-SNP member medical claims file (person-months in which individuals were enrolled in these plans) and Carrier (professional) claims file (person-months in fee-for-service Medicare) as follows:
  - For person-months in UPMC D-SNP/FIDE-SNP, we counted unique evaluation and management visits with either primary care clinicians or specialists. Measured from the UPMC Member Medical claims file where:
    - Place of service was a physician's office, hospital outpatient department, FQHC, or rural health clinic: (HIAA_POS_CODE) = (11, 22, 50, 72)
    - Service was an evaluation and management visit: CPT_PROCEDURE=(99201, 99202, 99203, 99204, 99205, 99211, 99212, 99213, 99214, 99215, G0402, G0438, G0439)
    - Claim line of business (CLAIM_LOB) = Medicare/SNP

    Counted unique outpatient evaluation and management visits by deduplicating records by person ID, service end date (SERVICE_END_DATE), and procedure code (CPT_PROCEDURE)
  - For person-months in fee-for-service Medicare, counted unique evaluation and management visits with either primary care clinicians or specialists. Measured from the FFS Carrier line file where:
    - Place of service was a physician's office, hospital outpatient department, FQHC, or rural health clinic: LINE_PLACE_OF_SRVC_CD = (11, 22, 50, 72)
    - Service was an evaluation and management visit: HCPCS_CD=(99201, 99202, 99203, 99204, 99205, 99211, 99212, 99213, 99214, 99215, G0402, G0438, G0439)

    Counted unique outpatient evaluation and management visits by deduplicating records by person ID, claim through date (CLM_THRU_DT), and HCPCS code (HCPCS_CD)
- Used the month of the service end date when aggregating counts to the person-month
  - Linked counts derived from UPMC Member Medical Claims files to all person-months when a person was enrolled in the D-SNP/FIDE-SNP.
  - Linked counts derived from FFS Carrier file to all person-months when a person was enrolled in FFS Medicare.

3. **Number of standardized 30-day drug fills of chronic disease medications[12] (count)**
   - We counted the number of standardized 30-day fills for chronic disease drugs per person-month. National Drug Codes (NDCs) were identified from a Medispan® crosswalk between drug classes and NDC codes:

Drug class	Medispan® codes (GPI Codes, which map to NDC codes based on annual crosswalk)
§ Statins	3940
§ Oral hypoglycemic agents and insulin	27

© 2023 Roberts ET et al. *JAMA Health Forum.*

§ Bronchodilators; anti-asthma drugs	44
§ Beta blockers; ace inhibitors; calcium channel blockers; antihypertensives and antihypertensive combinations; thiazides and related diuretics; loop diuretics; angiotensin inhibitors; alpha blockers; antianginals; platelet aggregation inhibitors	'32', '3310', '3320', '3330', '3610', '3400', '3760', '3720', '3625', '3615', '3617', '3640', '3660', '3699', '8515'
§ Antiglaucoma agents	'8633', '8680', '8625', '868023'
§ Antipsychotics; tricyclic antidepressants; monamine oxidase inhibitor antidepressants; phenothiazine/related antipsychotics; other antidepressants; benzodiazepines	'59', '58', '6299', '721000', '571000'

- We identified all drugs in the above-referenced subclasses from annual crosswalks of Medispan® codes to NDC codes. For drugs identified in these subclasses, we measured standardized 30-day fills from UPMC D-SNP/FIDE-SNP pharmacy claims file (person-months in which individuals were enrolled in these plans) and Part D event file (person-months in fee-for-service Medicare) as follows:
  - For person-months in UPMC D-SNP/FIDE-SNP, used the UPMC Member Pharmacy Claims file to total days' supply of drugs in the selected subclasses, divided by 30 (=Total days suppled [DAY_SUPPLY_CNT] in the subclasses/30)). Counted standardized 30-day fills by person-month (using fill date of prescription).
  - For person-months in FFS Medicare, used the Part D event file to total days' supply of drugs in the selected subclasses, divided by 30 (=Total days suppled [DAY_SUPPLY_CNT] in subclasses /30)). Counted standardized 30-day fills by person-month (using fill date of prescription).
- Used the month of the service through date when aggregating counts to the person-month
  - Linked counts derived from UPMC Member Pharmacy Claims files to all person-months when a person was enrolled in the D-SNP/FIDE-SNP.
  - Linked counts derived from Part D event file to all person-months when a person was enrolled in FFS Medicare.

4. **Hospital visits followed within 14 days by an outpatient visit[18] (proportion)**
   - We counted the proportion of a patient's hospital visits per month that were followed by an outpatient visit (evaluation and management service) within 14 days
   - Hospital visits were defined as inpatient admissions, emergency department visits that did not lead to an admission, and observation stays that did not lead to an admission
   - To construct this measure, we:

© 2023 Roberts ET et al. *JAMA Health Forum.*

a. Constructed a deduplicated longitudinal record of hospital visits and outpatient visits per person using UPMC member medical claims <u>and</u> fee-for-service claims files.
b. Sorted this longitudinal dataset by encrypted person ID, admission date/service start date, and discharge date/service end date/claim through date.
c. Flagged each index hospital stay with a 0/1 indicator to denote whether it was followed within 14 days by an outpatient visit for the same patient. We used the discharge date/end date of the hospital stay (Date2) to identify the month of the index hospital stay and the date of the outpatient visit (Date 1) to identify follow-up care. An example (hypothetical data for one patient) appears below:

Service	Date1	Date2	Follow-up within 14 days:
Inpatient admission	Admission date (1/17)	Discharge date (1/20)	0
ED visit	Service end date/claim through date (2/5)	Service end date/claim through date (2/5)	1
Outpatient visit	Service end date/claim through date (2/8)	Service end date/claim through date (2/8)	--

d. Counted number of index hospital visits per month that were followed by an outpatient evaluation and management visit within 14 days, and reported this as a proportion of index hospital visits per month that were followed up with an outpatient visit.
e. We dropped December 2020 because we did not have a complete 14-day follow up period for all index hospitalizations in this month.

5. **Inpatient care days per person-month[11,12,14-18] (days)**
- We counted the total number of days per person-month in which patients received inpatient care.
- Measured from UPMC D-SNP/FIDE-SNP member medical claims file (person-months in which individuals were enrolled in these plans) and MedPAR file (person-months in fee-for-service Medicare):
  - <u>For person-months in UPMC D-SNP/FIDE-SNP</u>, we measured inpatient admissions from the UPMC Member Medical Claims file where:
    - Claim type (CLAIM_TYPE) = ‘Acute inpatient facility/Inpatient Facility Medical & Surgical’
    - Claim line of business (CLAIM_LOB) = Medicare/SNP
    - Constructed a person-day record of days of inpatient care (the date of admission and date of discharge are both counted towards days of inpatient care)
  - <u>For person-months in FFS Medicare</u>, we measured inpatient admissions from the MedPAR file where:

© 2023 Roberts ET et al. *JAMA Health Forum.*

        - MedPAR Short Stay/Long Stay/SNF Indicator Code (SS_LS_SNF_IND_CD)=S
        - Constructed a person-day record of days of inpatient care (the date of admission and date of discharge are both counted towards days of inpatient care)
- Counted total days per person-month of inpatient care
    - Counted days of inpatient care from UPMC Member Medical Claims files for person-months when a person was enrolled in the D-SNP/FIDE-SNP.
    - Counted days of inpatient hospital care from FFS MedPAR file for person-months when a person was enrolled in fee-for-service Medicare.

6. **Inpatient admissions for ambulatory care-sensitive conditions[15-17] (count)**
    - We counted the number of inpatient admissions for ambulatory care-sensitive conditions per person-month.
    - Construction:
        a. Counted unique inpatient admissions from UPMC Member medical claims file (person-months in D-SNP/FIDE-SNP) and MedPAR file (person-months in fee-for-service Medicare). To count unique admissions, we combined admissions with any overlapping days for the same individual.
        b. Flagged admissions for ambulatory care-sensitive conditions based on the AHRQ Prevention Quality Indicators (PQI) algorithm (ICD-9 version for January 1, 2015-September 30, 2015, 1; ICD-10 version for October 1, 2015-present). AHRQ algorithm uses diagnoses fields on claims. We counted all admissions for ambulatory care-sensitive conditions based on the AHRQ Prevention Quality Indicators (PQI) algorithm (https://qualityindicators.ahrq.gov/measures/PQI_TechSpec).
        c. Use the month of the discharge date when aggregating counts to the person-month Use the month of the discharge date when aggregating counts to the person-month
            - Link counts derived from UPMC Member Medical Claims files to all person-months when a person was enrolled in the D-SNP/FIDE-SNP.
            - Link counts derived from the MedPAR file to all person-months when a person was enrolled in fee-for-service Medicare.

7. **Emergency department (ED) visits and observation stays[11,17,19] (count)**
    - We counted the number of ED visits and observation stays per person-month that did not lead to an inpatient admission
    - Measured from UPMC D-SNP/FIDE-SNP member medical claims file (person-months in which individuals were enrolled in these plans) and MedPAR file (person-months in fee-for-service Medicare):
        - For person-months in UPMC SNP, we counted unique ED visits and observation stays per person-month, limited to ED visits and observation stays that *did not* lead to an inpatient admission. Measured from the UPMC Member Medical claims file where:

© 2023 Roberts ET et al. *JAMA Health Forum.*

        - Claim type (CLAIM_TYPE) = ‘Emergency Room’ <u>OR</u> Revenue code (REVENUE_CODE) = (0450, 0451, 0452, 0456, 0459, 0760, 0761, 0762, 0769)
        - Claim line of business (CLAIM_LOB) = Medicare/SNP
        - The ED visit or observation stay did not occur during a patient’s inpatient hospital stay, including the admission date/discharge date of the inpatient stay. *We interpreted cases in which the ED visit/observation stay occurs on the same day of, or during the span of, an inpatient hospital stay as an ED visit/observation stay that led to an inpatient admission.* An example (hypothetical data for one patient) is below.
            - Count unique ED visits that do not occur during the span of an inpatient hospital stay.
            - ED visits or observation stays that have the same service begin date are counted as one visit.
    - <u>For person-months in FFS Medicare</u>, we counted unique ED visits and observation stays per person-month, limited to ED visits and observation stays that did not lead to an inpatient admission. Measured from the FFS outpatient revenue file where:
        - Revenue code (REV_CNTR) = (0450, 0451, 0452, 0456, 0459, 0760, 0761, 0762, 0769)
        - The ED visit or observation stay does not occur during an individual’s inpatient hospital stay, including the admission date/discharge date of the inpatient stay. An example is below
            - Count unique ED visits by deduplicating records (records meeting criteria 1 and 2) by person ID and the claim through date (CLAIM_FROM_DATE).
            - ED visits or observation stays that have the same claim from date or claim through date are counted as one visit
- Used the month of the service through date/end date when aggregating counts to the person-month.
    - Linked counts derived from UPMC Member Medical Claims files to all person-months when a person was enrolled in the SNP.
    - Linked counts derived from FFS MedPAR file to all person-months when a person was enrolled in FFS Medicare.
- Illustration of how we counted emergency department visits and observation stays that did not lead to an inpatient admission (hypothetical data for one patient):

**Type of visit**	**Start date (service begin date for ED or inpatient admission date)**
ED	1/1/2020
~~ED~~	~~2/1/2020~~ (because this ED visit occurred on the same date as an inpatient admission, we consider it to be an ED visit that led to an admission)
Inpatient admission	2/1/2020
Observation stay	6/1/2020

© 2023 Roberts ET et al. *JAMA Health Forum.*

8. **Post-acute Skilled Nursing Facility (SNF) care days (Medicare-covered) (days)**
   - We counted the total number of days per person-month in which individuals received Medicare-covered skilled nursing facility care (for post-acute care).
   - Measured from UPMC D-SNP/FIDE-SNP member medical claims file (person-months in which individuals were enrolled in these plans) and MedPAR file (person-months in fee-for-service Medicare):
     - For person-months in UPMC D-SNP/FIDE-SNP, we measured SNF stays from the UPMC Member Medical Claims file where:
       - Revenue codes corresponded to skilled nursing facility stays (REVENUE_CODE = 0022, 0110, 0120)
       - Constructed a person-day record of days of SNF care (date of admission and date of discharge are counted towards days in SNF)
     - For person-months in FFS Medicare, we measured SNF stays from the MedPAR file where:
       - MEDPAR Short Stay/Long Stay/SNF Indicator Code (SS_LS_SNF_IND_CD)=N
       - Constructed a person-day record of days of SNF care (date of admission and date of discharge are counted towards days in SNF)
   - Counted total days per person-month of SNF care
     - Counted days of SNF care from UPMC Member Medical Claims files for person-months when a person was enrolled in the D-SNP/FIDE-SNP.
     - Counted days of SNF care from FFS MedPAR file for person-months when a person was enrolled in fee-for-service Medicare.

9. **Post-acute home health care days (Medicare-covered) (days)**
   - We counted total number of days per person-month in which individuals received Medicare-covered home health care (for post-acute care).
   - For person-months in UPMC SNP, we counted unique person-days per person-month of home health services. Measured from the UPMC Member Medical claims file where (i) OR (ii) is true:
     i. (HIPAA_POS_CODE=12) AND (CPT_PROCEDURE = (G0151, G0152, G0153, G0154, G0155, G0156, G0157, G0158, G0159, G0160, G0161, G0162, G0163, G0164, G0299, G0300, G0493, G0494, G0495, G0496, Q5001, Q5002, Q5009)
     ii. Revenue center code (REVENUE_CODE) = (042*, 043*, 044*, 055*, 056*, 057*)
     * = wildcard
     - In both (i) and (ii), limit to claim line of business (CLAIM_LOB) = Medicare/SNP
       - Unique person-days per month calculated by deduplicating records to the person ID and service end date (SERVICE_END_DATE)
       - Report count of unique person-days per month
   - For person-months in FFS Medicare, we counted unique person-days of home health services per person-month from the Home Health Agency (HHA) revenue center file and Carrier files as follows:

        - In HHA file: HCPCS Code (HCPCS_CD) = (G0151, G0152, G0153, G0154, G0155, G0156, G0157, G0158, G0159, G0160, G0161, G0162, G0163, G0164, G0299, G0300, G0493, G0494, G0495, G0496, Q5001, Q5002, Q5009) OR Revenue center code (REV_CNTR) = (042*, 043*, 044*, 055*, 056*, 057*).
          * = wildcard
        - In Carrier file: Place of service = 12 AND HCPCS Code (HCPCS_CD) = (G0151, G0152, G0153, G0154, G0155, G0156, G0157, G0158, G0159, G0160, G0161, G0162, G0163, G0164, G0299, G0300, G0493, G0494, G0495, G0496, Q5001, Q5002, Q5009) OR Revenue center code (REV_CNTR) = (042*, 043*, 044*, 055*, 056*, 057*).
          * = wildcard
            - Stack resulting records from the HHA and Carrier file
            - Count unique person-days per month calculated by deduplicating records in this stacked file to the person ID and claim through date (CLM_THRU_DT)
            - Report count of days of home health service use per person-month
- Used the month of the service end date/claim through date when aggregating counts of days to the person-month.
    - Linked counts from UPMC Member Medical Claims files to all person-months when a person was enrolled in the D-SNP/FIDE-SNP.
    - Linked counts derived from FFS HHA/Carrier files to all person-months when a person was enrolled in FFS Medicare.

10. Days of long-term nursing home care[11,12,14-16] (days)

- We counted the number of unique days per person-month in which individuals received Medicaid-covered long-term nursing home care during the period 2017-2020 (provided for long-stay nursing home residents).
    - 2018-2020 outcomes measured from UPMC Medicaid managed care files
    - 2017 is baseline, measured from Medicaid service history file (for Pennsylvania's predecessor fee-for-service Medicaid program).
        - We dropped data from the first quarter of 2017 to allow for a sufficient run-in period to measure new nursing home admissions (admissions beginning prior to 2017 and continuing into 2017 were not fully captured in the service history file).
        - We measured days of long-term nursing home care (not admissions) because long-term nursing home stays tend to extend beyond one month.
- Measured from the UPMC Member Medical claims file/Medicaid service history file where:
    - Revenue code for long-term nursing home stays (REVENUE_CODE = 0100)
    - In UPMC Member Medical claims, records where line of business (CLAIM_LOB) = CHC or Medicaid (MA)
- Counted number of unique days of long-term nursing home care per person-month

© 2023 Roberts ET et al. *JAMA Health Forum.*

Covariates

We included the following covariates from administrative data. Covariates were measured in the baseline (pre-integration) period; means and proportions of these covariates are shown in Table 1 of the main manuscript. These covariates were included in the propensity score and outcome models in our main analyses, except for receiving >100 days of long-term nursing home care in 2017, which differed substantially across the integration and comparison cohorts at baseline. To explore the impact of baseline differences in long-term nursing home care on our findings, we conducted a sensitivity analysis that excluded long-term nursing home residents in 2017 from both the integration and comparison cohorts.

Covariates were defined as follows:

- **Age in 2017,** categorized as follows: <50 years, ≥50 to ≤54 years, ≥55 to ≤59 years, ≥60 to ≤64 years, ≥65 to ≤74 years, ≥75 to ≤79 years, ≥80 years. (For brevity, descriptive tables in the main manuscript report mean age only rather than the distributions of individuals across these age categories.)
- **Sex,** measured from administrative enrollment data and categorized as male, female, or unknown. We do not separately report the share of subjects with unknown sex due to the very small number of observations in this category.
- **Race and ethnicity,** measured from administrative enrollment data. We categorized race and ethnicity into six mutually exclusive groups: 1) non-Hispanic White, 2) Non-Hispanic Black/African American, 3) Hispanic, 4) Asian or Pacific Islander, 5) American Indian or Alaska Native, 6) or another race. Our propensity score and outcome regression models included a 6-level categorical race and ethnicity variable. However, because of small sample sizes, we pooled the fourth

© 2023 Roberts ET et al. *JAMA Health Forum.*

through sixth categories into one group when reporting the race and ethnicity distributions of study cohorts in the main manuscript.

- **Disabled,** measured from the "original reason for Medicare entitlement" (OREC) variable in administrative enrollment data. The OREC variable indicates whether a person originally qualified for Medicare because of a disability vs. age.
- **Elixhauser comorbidity index.** This index is a count of 31 comorbidity categories and is based on the Elixhauser algorithm for categorizing comorbidities in administrative data into clinically relevant domains that predict utilization, spending and mortality.[20] We constructed this index from diagnoses in administrative Medicare data in 2017 (UPMC data for the integration cohort and traditional Medicare data for the comparison cohort). We used Medicare claims as Medicare is the primary payer for inpatient, outpatient, and most professional services for dual eligible individuals, where diagnoses for comorbid conditions will be captured. In the propensity score and outcome models, we included the index as a continuous variable. To account for threshold effects associated with having multiple comorbidities, we also included indicators for having $\geq$5 and $\geq$10 comorbidities.
- **Area Deprivation Index (ADI).** The ADI is derived from 17 Census block-level socioeconomic and demographic variables and reflect a community's level of socioeconomic disadvantage.[9] We categorized the ADI into quintiles based on the national distribution of this index, and linked quintiles to study subjects using a crosswalk between Census block and 9-digit ZIP code (the latter was reported in administrative enrollment files).We used each subject's ZIP code of residence in

© 2023 Roberts ET et al. *JAMA Health Forum.*

2017 for this linkage. We included indicators of ADI quintiles in propensity score and outcome models. For brevity, descriptive tables show the proportions of dual eligible individuals living in communities with a high degree of socioeconomic deprivation (quintiles 4 or 5 of the ADI) versus lower level of deprivation (quintiles 1-3).

- **Rural residence,** defined using primary Rural-Urban Commuting Area (RUCA) codes published by USDA.[10] We linked primary RUCA codes to each study subject's ZIP code of residence (5-digit ZIP code) in 2017. Urban areas were defined as those with primary RUCA codes ≤ 3; rural areas were considered those with primary RUCA ≥4 to ≤10.

© 2023 Roberts ET et al. *JAMA Health Forum.*

V. Statistical Analyses

We used a difference-in-differences design to compare changes in outcomes between the integration versus comparison cohorts, from a baseline period preceding the availability of integrated coverage (2015-2017), through the first three years of integration in UPMC-operated Medicare and Medicaid managed care plans (2018, 2018, and 2020), and contemporaneous periods in the comparison cohort. We conducted analyses in two stages, as follows.

First, we constructed propensity score weights to weight the comparison cohort to resemble the integration cohort on baseline characteristics (measured in 2017). To construct these weights, we estimated a logistic regression model predicting membership in the integration cohort as a function of baseline covariates described in Section IV. For each person in the comparison cohort, we constructed the time-invariant propensity score weight $\widehat{p_i(x)}/(1 - \widehat{p_i(x)})$, where $\widehat{p_i(x)}$ denotes the estimated propensity score for subject *i*. Each person in the integration cohort was assigned a weight of 1. This approach, termed Average Treatment Effect on Treated (ATT) weighting, weights the comparison cohort to resemble the integration cohort on baseline characteristics.[21]

Second, we estimated propensity score-weighted difference-in-differences models to compare changes in outcomes between the integration vs. comparison cohorts. For each outcome, y_{it}, we estimated a linear difference-in-differences model of the form:

$$y_{it} = \beta_0 + \beta_1 \text{Integrate}_i + \beta_2 \text{Integrate}_i \times 2018_t + \beta_3 \text{Integrate}_i \times 2019_t + \beta_4 \text{Integrate}_i \times 2020_t + \beta_5 X_i + \text{year}_t + \text{quarter}_t + \varepsilon_{it} \quad (1)$$

The unit of analysis was the person-month. Above, Integrate_i denotes membership in the integration cohort; 2018_t, 2019_t, and 2020_t are indicators for the three post-integration years; X_i are covariates measured at baseline (described in Section IV); year_t and quarter_t are year and

© 2023 Roberts ET et al. *JAMA Health Forum.*

quarter fixed effects, respectively (2017 was omitted as the reference); and ε_{it} is a heteroskedacity-robust error term clustered at the person level. Thus, β_2, β_3, and β_4 represent adjusted differential changes in outcome y in the integration vs. comparison cohorts from the baseline period through the first, second, and third years of integration (respectively). For example, where the study outcome is days of HCBS use per person-month, β_2 represents the adjusted differential change (comparing the integration vs. comparison cohorts) in HCBS days per person-month from baseline to 2018.

Assumptions of the difference-in-differences design

The central assumption of our difference-in-differences design is that the comparison cohort controls for trends in outcomes that would have been expected in the integration cohort had UPMC's D-SNP not become integrated with a Medicaid managed care plan. (Stated differently, the comparison cohort is a plausible counterfactual for trends that would be expected without integration, such as changes linked only to the implementation of Community HealthChoices, which affected both cohorts.) Consequently, the validity of our design hinges on the assumptions that 1) there were no baseline differences between the cohorts that would have predisposed them to follow different outcome trends without integration, and 2) unmeasured time-varying factors did not differentially affect the cohorts in the post-integration period. Our analysis of a within-state comparison group of dual eligible individuals, which we weighted to resemble the integration cohort on baseline characteristics, was designed to support the plausibility of these assumptions. However, there remains the potential for confounding from unmeasured factors that would predispose the cohorts to follow different trends past 2018 without integration.

© 2023 Roberts ET et al. *JAMA Health Forum.*

A standard way of testing for such bias is to examine whether baseline outcome trends were "parallel" between integration and comparison cohorts. Parallel trends in the baseline period suggest the cohorts likely would have continued to follow similar outcome trajectories past 2018 without integration.[22] We tested this assumption by estimating linear event-study models to compare quarterly changes in outcomes across the integration and comparison cohorts relative to the last pre-integration quarter (quarter 4 of 2017, the reference). These models had the following form (unit of analysis = person-month):

$$y_{it} = \beta_0 + \beta_1 \text{Integrate}_i + \beta_2 X_i + \sum_{t \neq Q4:2017} \eta_t \, I(\text{quarter}_t = t) + \sum_{t \neq Q4:2017} \delta_t \, I(\text{quarter}_t = t) \times \text{Integrate}_i + \varepsilon_{it} \quad (2)$$

Above, δ_t represents a differential change (comparing the integration vs. comparison cohorts) in an outcome from the reference quarter to quarter *t*. (Quarter 4 of 2017, the last pre-integration quarter, was the reference quarter.) In eFigure 4, we plotted estimates of δ_t (and associated 95% CIs) to visualize quarterly trend differences between the integration and comparison cohorts. The parallel pre-trends assumption is violated when there is a clear pattern of δ_t differing from zero in the pre-intervention period. Differential pre-trends suggest we cannot confidently attribute differential changes after 2018 to the effects of integration, without imposing potentially strong assumptions on how different pre-trends would have continued to evolve without integration.[23] Event-study models were adjusted for covariates described in the methods and weighted by propensity score weights.

The parallel trends test is an important check on assumptions of the difference-in-differences design. However, in our setting, the parallel trends test may have limitations detecting differences in some outcome trends in the absence of integration. One reason for this limitation is that our cohort definitions required dual eligible individuals to have survived to

© 2023 Roberts ET et al. *JAMA Health Forum.*

January 2018, so that we could observe enrollment in UPMC's CHC Medicaid managed care plan (established in 2018). For outcomes that reflect survival or that are measured among dual eligible individuals at high risk of mortality (e.g., those receiving care in a nursing home), baseline trends may appear parallel across cohorts *because* we restrict the sample to survivors over the entire pre-integration period. However, differential changes in outcomes after 2018 may not reflect effects of integration, but instead unmeasured variables related to mortality risk factors, which become visible only after we remove the "constraint" on survival following January 2018.

Figure 1 in the main manuscript illustrates this issue. Survival rates in the integration and comparison cohorts are 100% from the baseline period through January 2018 by construction. However, *immediately after January 2018,* survival rates diverge, with a noticeably lower rate of survival in the comparison vs. integration cohorts. By December 2018—just one year after integration—the survival rate was 5.3 percentage points lower in the comparison vs. integration cohorts. We view such immediate survival differences as unlikely due to plan effects on health. Instead, these differences are likely a function of unmeasured risk factors that differed between the cohorts at baseline, whose link to mortality risk could not observe prior to 2018 because of how the cohorts were constructed. These survival differences contribute to differential attrition across the cohorts in the post-integration period, which in turn may affect our difference-in-differences estimates—particularly for outcomes in populations that experience a high risk of mortality (e.g., nursing home residents). eFigure5 plots these survival differences across cohorts, stratified according to whether dual eligible individuals in 2017 resided in a nursing home, lived in the community and enrolled in an HCBS waiver program, or lived in the community and did were not in an HCBS waiver program. The figure shows that survival rates were lower among

© 2023 Roberts ET et al. *JAMA Health Forum.*

dual eligible individuals who were nursing home residents at baseline, among particularly among those in the comparison vs. integration cohorts.

We conducted an analysis to examine how differences in attrition due to mortality in the post-period could have affected our estimates. Specifically, we compared difference-in-differences estimates of year-one (2018) effects before and after we excluded decedents in 2018 *from all study periods*. Appreciable changes in year-one effects when we do vs. do not exclude 2018 decedents from all periods suggest that findings were sensitive to survival differences, which were likely linked to unmeasured risk factors that preceded integration. These analyses are reported in Figure 2 of the main manuscript and in eFigure 6.

Supplementary analyses

We also conducted several supplementary analyses:

1) Because there were sizable differences in the receipt of long-term nursing home care at baseline (2017), we conducted a sensitivity analysis in the subsample of dual eligible individuals who in 2017 lived in the community (i.e., excluding long-term nursing home residents). We re-estimated propensity score models to balance observed characteristics of integration and comparison cohort members in this subsample.
2) We further stratified our difference-in-differences models to obtain separate estimates for three groups: a) individuals who in 2017 (baseline) were long-term nursing facility residents, b) those enrolled in an HCBS waiver program (for people living in the community who require a nursing facility level of care) in 2017, and c) those living in the community and not enrolled in an HCBS waiver program in 2017. To handle a small number of cases where individuals were included in multiple eligibility categories in 2017, we assigned categories hierarchically: first to the long-term nursing facility resident

category (if any long-term nursing facility residence was reported for 2017), second to the HCBS waiver category, and third to the community-dwelling and HCBS-ineligible category. Estimates from these models are reported in eTable 6.

A weighted average of these stratum-specific estimates produces the estimates in Table 2 of the main manuscript, where the weights are proportional to the number of survivors in each stratum each month (utilization is measured among survivors). However, these weights change over time due to differences in survivorship across the strata. Specifically, because mortality was highest in the stratum of dual eligible individuals who were long-term nursing home residents at baseline (particularly in the comparison group; eFigure 5), in the post-integration period, the overall average was weighted *less* towards dual eligible individuals who were nursing home residents at baseline and *more* towards dual eligible individuals living in the community. This results in our overall estimate of a greater decline in long-term nursing home stays in the *comparison* vs. *integration* groups over the post-integration period (corresponding to a positive and statistically significant difference-in-differences estimate for this outcome in Table 2), even when the difference-in-differences estimates in each stratum were small and nonsignificant. Because difference-in-differences estimates for long-term nursing home stays were driven by a compositional change in who remained alive during the post-integration period, and these differences in survival likely reflected the influence of unmeasured risk factors preceding integration, we do not interpret estimates for nursing home stays as causal effects of integration.

3) From 2018-2020, we examined changes in the proportions of dual eligible individuals who were classified as eligible for HCBS, which we analyzed separately among those

© 2023 Roberts ET et al. *JAMA Health Forum.*

who lived in the community and were HCBS-ineligible vs. HCBS-eligible in 2017. We also examined changes in long-term nursing facility eligibility among dual eligible individuals who had lived in the community and were HCBS-eligible in 2017. We focused on these outcomes because they reflect important transitions in eligibility for long-term care. They allow us to consider how having one insurer manage Medicare and Medicaid services for the same people might have influenced referrals for independent clinical assessments that resulted in changes in eligibility for different long-term services and supports.

Because we lacked person-month-level data for these variables prior to 2018, we descriptively characterized these changes for the 2018-2020 period, conditional on baseline HCBS eligibility in 2017 (we had annual person-level records for 2017). We estimated the following linear probability models:

$$E[C_{it}|Init_{i,2017}] = \beta_0 + \beta_1 Integrate_i + \sum_{t\neq Jan:2018} \eta_t I(month_t = t) + \sum_{t\neq Jan:2018} \delta_t I(quarter_t = t) \times Integrate_i \quad (3)$$

Above, C_{it} denotes a classification (i.e., community-dwelling HCBS-eligible or nursing facility-eligible) for person *i* in month *t* (observed from 2018-2020), and $Init_{i,2017}$ denotes person *i*'s initial HCBS eligibility in 2017 (i.e., community-dwelling and HCBS-ineligible, community-dwelling HCBS-eligible). Estimates of η_t capture monthly changes (relative to January 2018) in the comparison cohort and estimates of δ_t represent the difference in monthly changes (relative to January 2018) between the integration vs. comparison cohorts. We plotted η_t and $\eta_t + \delta_t$ to characterize trends from 2018-2020 in the two cohorts (eFigure 7). To examine whether differential mortality across cohorts affects this comparison of trends, we repeated analyses excluding decedents from 2018-2020.

© 2023 Roberts ET et al. *JAMA Health Forum.*

VI. Results

This section reports the following results:

1) Difference-in-differences estimates of changes in care associated with integrating Medicare and Medicaid coverage, which are our primary study estimates (eTable 3).

2) Plots of estimates from event-study models, in which we compared quarterly trends in study outcomes between the integration vs. comparison cohorts over the study window, relative to the 4th quarter of 2017 (the reference quarter) (eFigure 4).

3) Kaplan-Meier plots comparing survival rates in the integration and comparison cohort from 2018-2020, stratified according to whether dual eligible individuals in 2017 resided in a nursing home, lived in the community and enrolled in an HCBS waiver program, or lived in the community and were not in an HCBS waiver program (eFigure 5).

4) Analyses that examined differential outcome trends from baseline through 2018, under two scenarios: one that excluded decedents in 2018 from all periods, and a second that kept these decedents in all periods (eFigure 6). Each plot is constructed from an event-study regression analogous to model (2) but is run through 2018. These analyses allowed us to examine the impact of differential sample attrition due to mortality on estimates of year-one differential changes in outcomes associated with integration.

5) Analyses conducted among dual eligible individuals who in 2017 lived in the community (i.e., excluding those living in a nursing facility in 2017). We reported baseline characteristics of this sample before and after propensity score weighting (eTable 4) and difference-in-differences estimates for this subsample (Table 3 in the main manuscript).

© 2023 Roberts ET et al. *JAMA Health Forum.*

6) Analyses in which we further stratified difference-in-differences models according whether dual eligible individuals in 2017 resided in a nursing facility, lived in the community and qualified for an HCBS waiver program, or lived in the community and were ineligible for an HCBS waiver program (eTable 5).
7) Monthly changes in HCBS eligibility (analyzed separately among community-dwelling duals who were HCBS-ineligible vs. HCBS-eligible in 2017) and eligibility for long—term nursing facility care (among duals who were community-dwelling and HCBS-eligible in 2017) (eFigure 7).

© 2023 Roberts ET et al. *JAMA Health Forum.*

eTable 3: Difference-in-differences estimates of changes in care associated with integrating Medicare and Medicaid coverage

Outcome [a,b]	Mean of outcome in integration cohort in 2017	Differential change from baseline to: Year one of integration (2018)	Year two of integration (2019)	Year three of integration (2020)
		Difference-in-differences estimate (95% CI)		
Receipt of home and community-based services (HCBS) [c]	2.83	0.28*** (0.10, 0.46)	0.49**** (0.22, 0.77)	0.61**** (0.28, 0.94)
Care management and coordination:				
Outpatient visits	0.74	-0.02 (-0.06, 0.02)	-0.05 (-0.11, 0.01)	-0.12*** (-0.17, -0.07)
Standardized 30-day fills of chronic disease medications	3.34	0.01 (-0.04, 0.07)	-0.05 (-0.12, 0.02)	-0.02 (-0.10, 0.06)
Hospital visit followed within 14 days by an outpatient visit	0.47	0.00 (-0.04, 0.04)	0.02 (-0.01, 0.05)	-0.01 (-0.04, 0.03)
Hospital and post-acute care use:				
Inpatient care	0.16	0.00 (-0.04, 0.03)	-0.02 (-0.06, 0.03)	0.03 (-0.01, 0.07)
Inpatient admissions for ambulatory care-sensitive conditions	0.01	0.00 (-0.01, 0.01)	0.00 (-0.01, 0.01)	0.00 (-0.01, 0.01)
Emergency department visits and observation stays	0.10	0.00 (-0.01, 0.01)	-0.01 (-0.02, 0.00)	-0.01 (-0.03, 0.01)
Post-acute skilled nursing facility care	0.23	0.06 (-0.01, 0.12)	0.07* (0.00, 0.13)	0.03 (-0.03, 0.08)
Post-acute home health care	1.00	0.14* (0.00, 0.28)	0.13 (-0.02, 0.29)	0.32**** (0.15, 0.49)
Long-term nursing facility use:				
Long-term nursing home care [c]	1.10	0.10* (-0.01, 0.21)	0.52*** (0.25, 0.80)	1.06*** (0.74, 1.38)

P-values denoted as follows: * P<0.1, ** P<0.05, *** P<0.01, **** P<0.001.

[a] Table reports difference-in-differences estimates, which are differential changes in outcome between the integration and comparison cohorts from baseline through three years of integration (2018, 2019, and 2020). The unit of analysis is the person-month. The baseline period is 2015-2017 for all outcomes except

© 2023 Roberts ET et al. *JAMA Health Forum.*

those measured from Medicaid claims, where the baseline period was limited to 2017 due to available data (these outcomes are indicated by footnote c). Estimates adjusted for covariates described in methods and weighted by propensity score weights. In this analysis, covariates in the propensity score and outcome models include receipt of more than 100 days of nursing facility care in 2017 as a covariate (inclusion of this variable in the propensity score model balances on this covariate). P-values reflect statistical significance of differential changes between the integration versus comparison cohorts from baseline through each post-integration year.
[b] See the notes to Table 2 of the main manuscript for descriptions of the outcome variables.
[c] Measured from Medicaid claims.

© 2023 Roberts ET et al. *JAMA Health Forum.*

eFigure 4: Estimates from event-study models

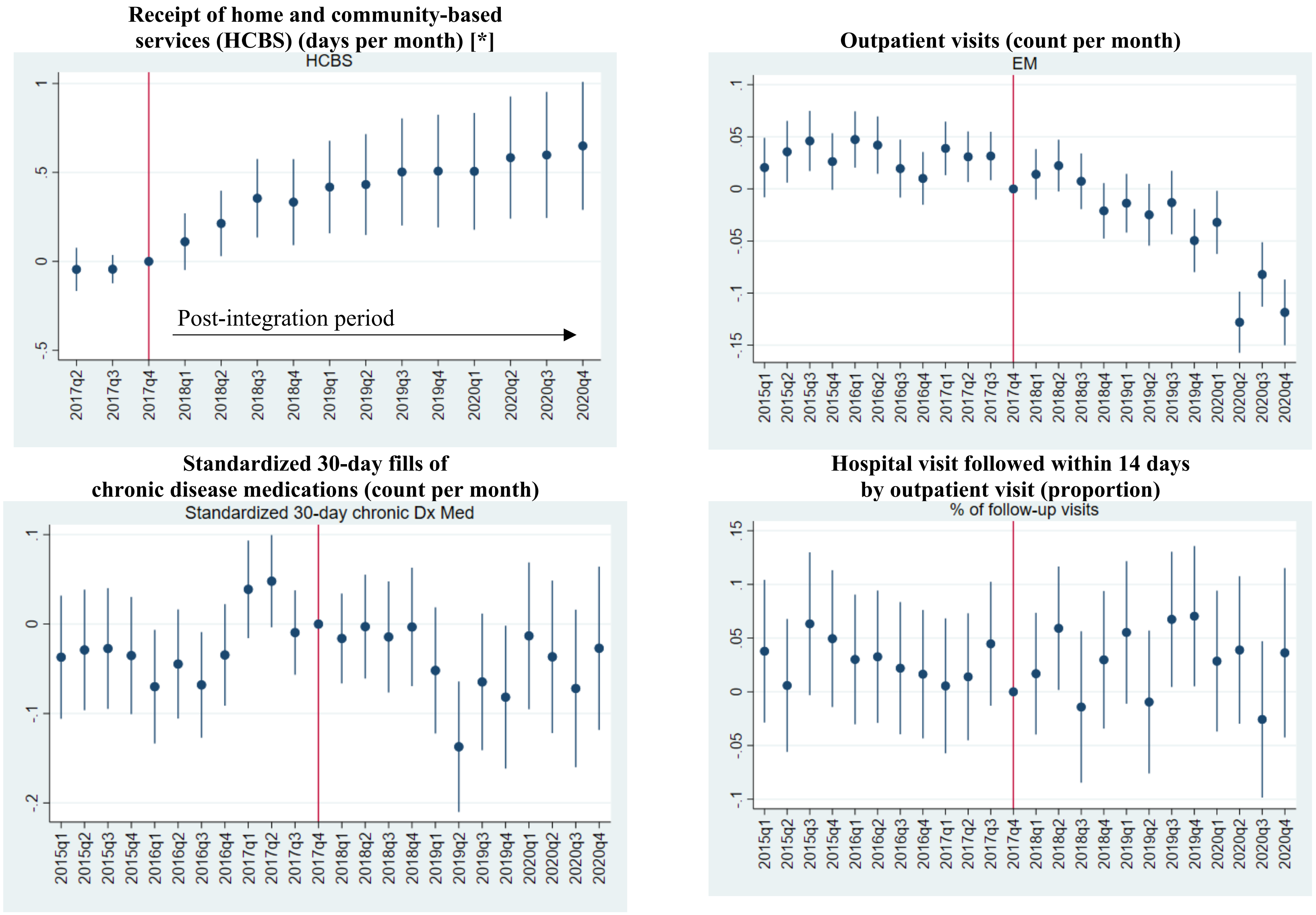

© 2023 Roberts ET et al. *JAMA Health Forum.*

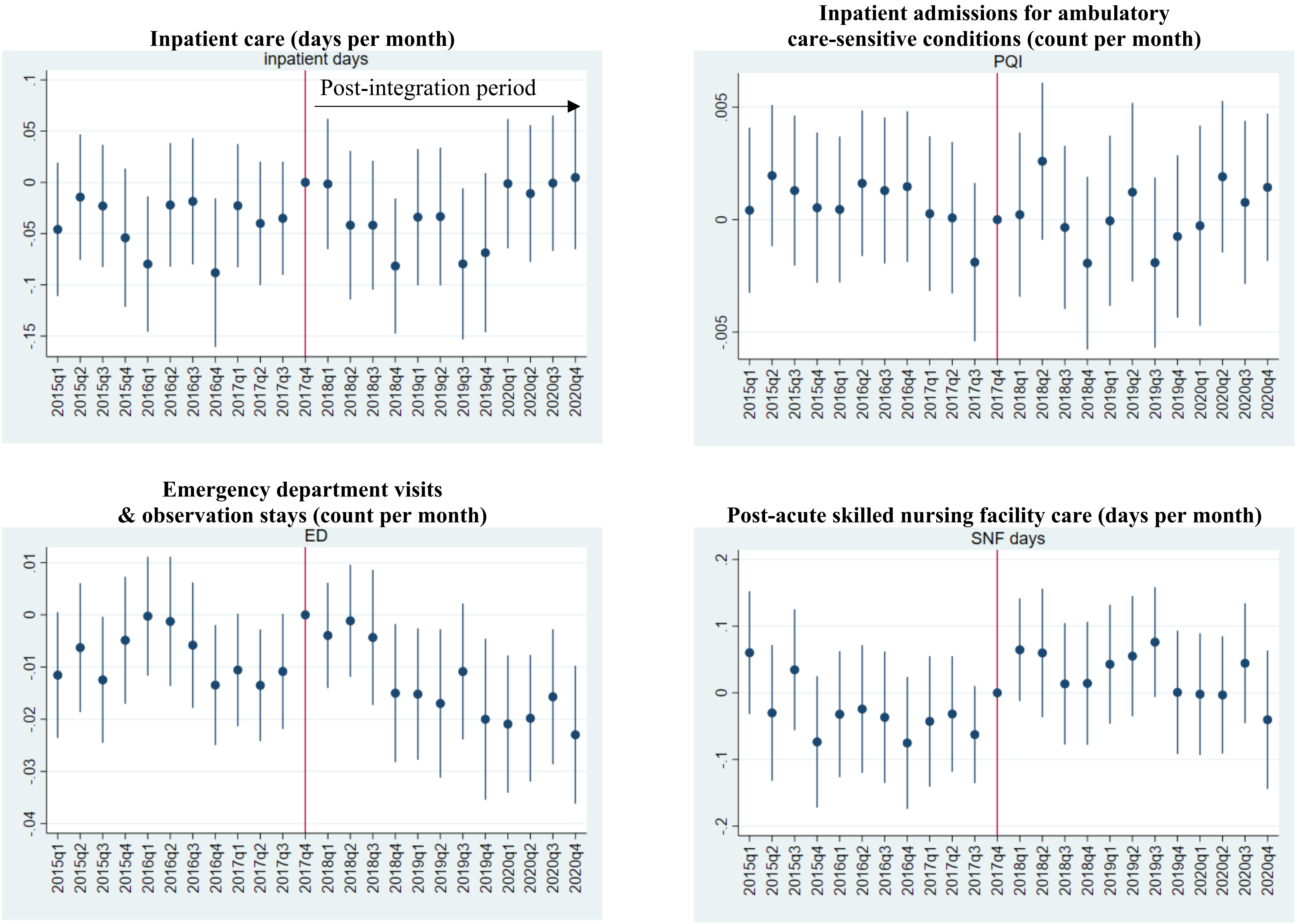

© 2023 Roberts ET et al. *JAMA Health Forum.*

Post-acute home health care (days per month)

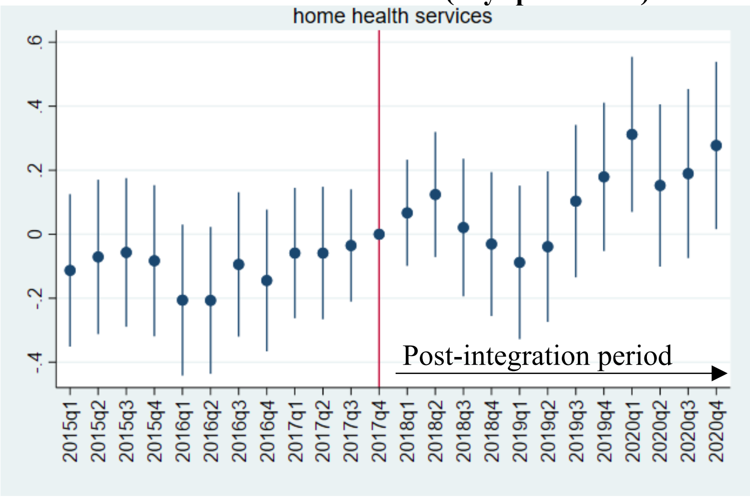

Long-term nursing home care (days per month) [*]

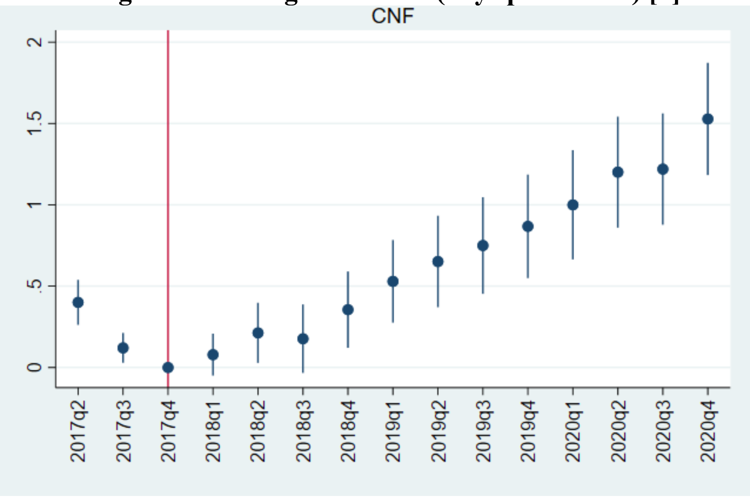

Plots show estimates of δ_t (and associated 95% CIs) from event study models (see equation 2), which compare outcome trends in the integration vs. comparison cohorts relative to the 4th quarter of 2017 (reference period). We plotted estimates of δ_t to visualize differences in quarterly trends between the integration and comparison cohorts. When δ_t is statistically indistinguishable from zero in the baseline period (prior to 2018), this provides support of the parallel baseline trends assumption. Estimates adjusted for covariates described in methods and weighted by propensity score weights. The baseline period is 2015-2017 for all outcomes except those measured from Medicaid claims (marked by an asterisk [*]), where the baseline period was limited to 2017 due to available data.

© 2023 Roberts ET et al. *JAMA Health Forum.*

eFigure 5: Survival plots stratified according whether dual eligible individuals in 2017 resided in a nursing home, lived in the community and were enrolled in an HCBS waiver program, or lived in the community and were not in an HCBS waiver program

Stratum: resided in a nursing home in 2017

(n=328 in integration cohort; n=985 in comparison cohort)

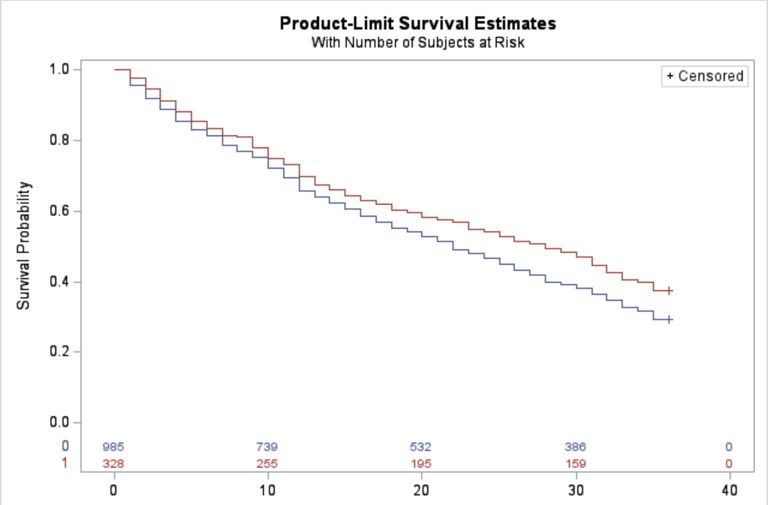

Time (months in post-integration period [up to 36 months])

Stratum: lived in the community and enrolled in an HCBS waiver program in 2017

(n=988 in integration cohort; n=448 in comparison cohort)

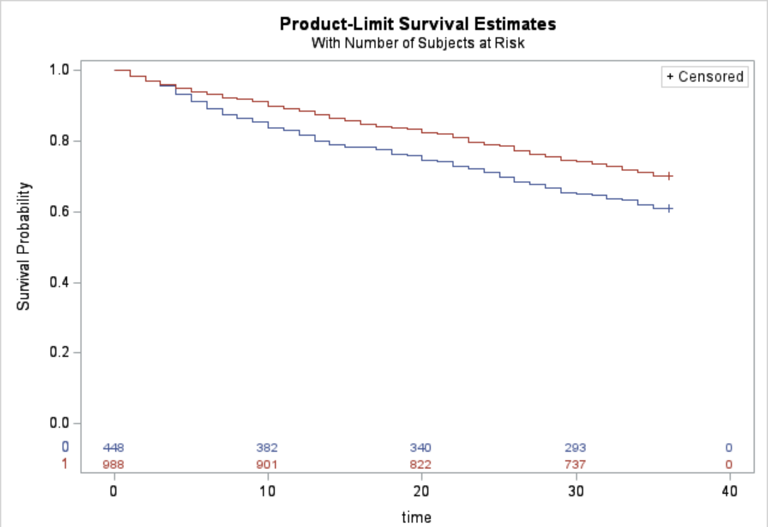

Time (months in post-integration period [up to 36 months])

© 2023 Roberts ET et al. *JAMA Health Forum.*

Stratum: lived in the community and not in an HCBS waiver program in 2017
(n=6,651 in integration cohort; n=2,399 in comparison cohort)

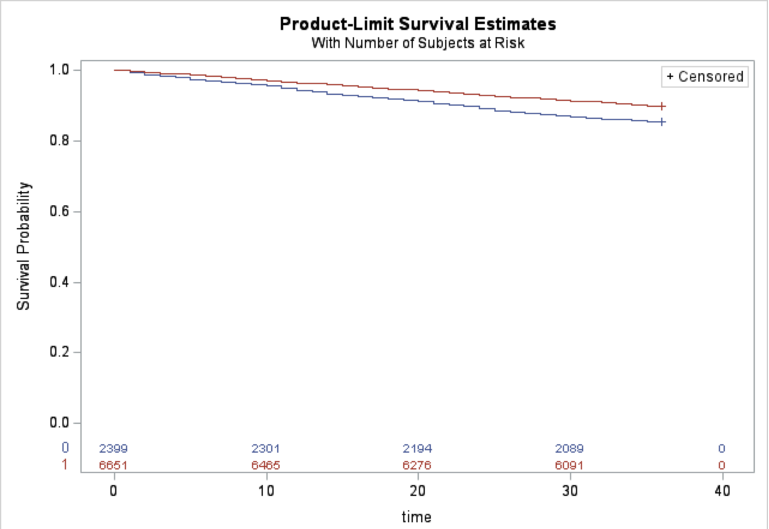

Time (months in post-integration period [up to 36 months])

Legend:
Integration cohort
Comparison cohort

Graphs are Kaplan-Meier survival plots, which depict the proportion of dual eligible individuals in the integration and comparison cohorts who remained alive by each post-integration month. We analyzed dates of death reported for decedents in the integration and comparison cohorts. Individuals who remained alive to the end of the post-integration period (36 months) are analyzed as censored.

© 2023 Roberts ET et al. *JAMA Health Forum.*

eFigure 6: Comparison of adjusted trends in outcomes in the integration and comparison cohorts, 2015-2018, before and after excluding decedents in 2018

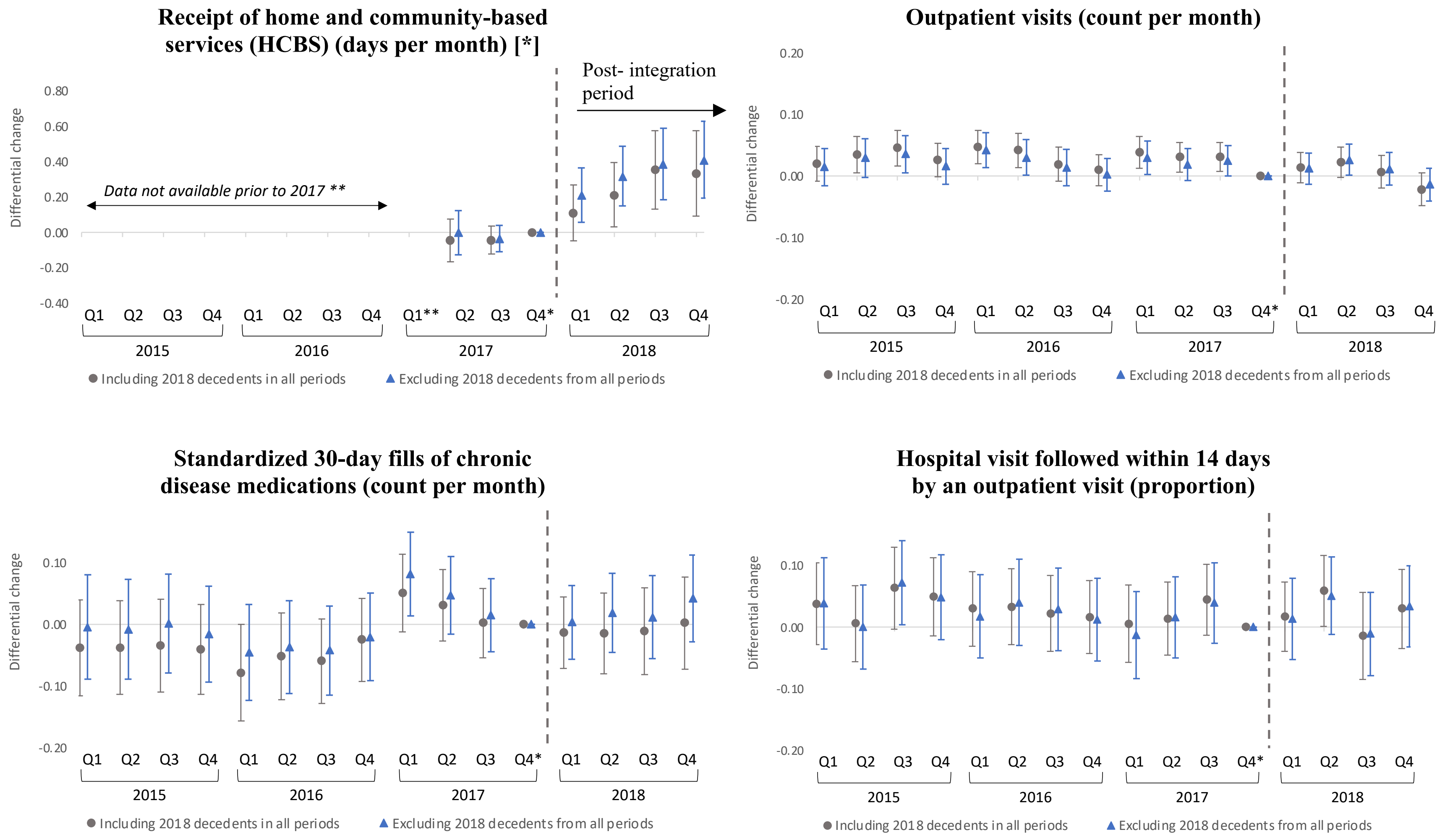

© 2023 Roberts ET et al. *JAMA Health Forum.*

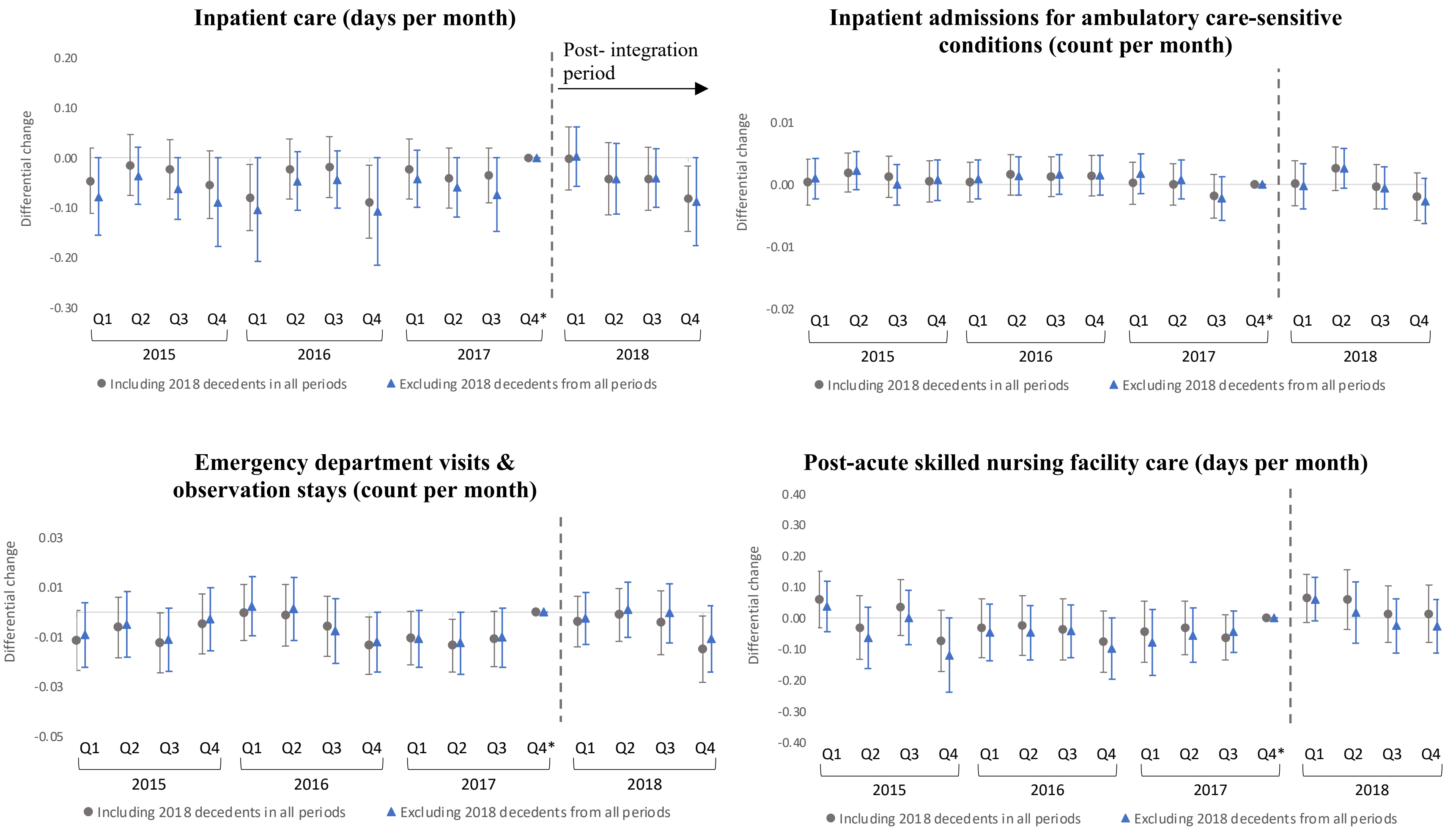

© 2023 Roberts ET et al. *JAMA Health Forum.*

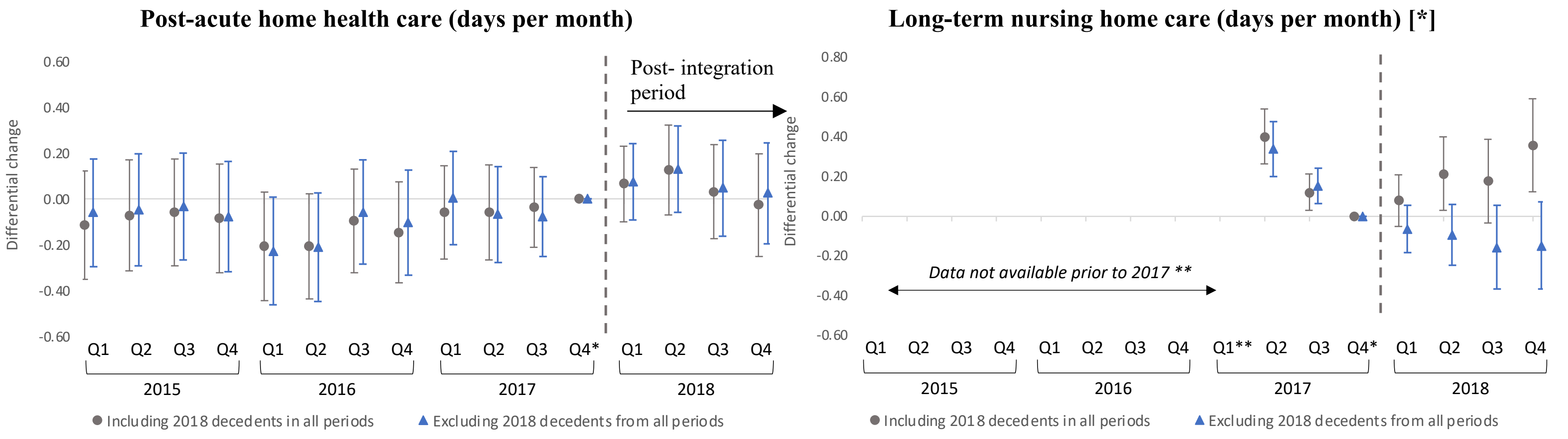

Each graph displays the adjusted differential change in the outcome by quarter in the integration versus comparison cohorts, relative to the first quarter of 2017 (the reference period). Estimates obtained from event-study models, which were adjusted for covariates and weighted by propensity score weights. Whiskers around each annual estimate give the 95% confidence interval for that estimate. Grey dots denote estimates from our main analysis sample, which included decedents in the post-integration period. Blue triangles denote estimates from a sensitivity analysis sample, which excluded decedents in 2018 from all periods. Dashed vertical bars distinguish the pre-integration period (2015-2017) and the post-integration period (2018). The baseline period is 2015-2017 for days of HCBS use and days of long-term nursing facility care (both measured in Medicaid claims) where the baseline period was limited to 2017 due to available data (indicated by [*] in the figure).

© 2023 Roberts ET et al. *JAMA Health Forum.*

eTable 4: Characteristics of sensitivity analysis sample: dual eligible individuals living in the community at baseline (2017)

	Before propensity score weighting			After propensity score weighting		
	**Integration Cohort (n=7,639) [b]**	**Comparison Cohort (n=2,847) [b]**	**Standardized mean difference [c]**	**Integration Cohort (n=7,639) [b]**	**Comparison Cohort (n=2,847) [b]**	**Standardized mean difference [c]**
**Covariates: [a]**						
Mean age in 2017 (years)	62.7	59.0	0.24	62.7	62.1	0.03
Female sex, %	65.8%	57.3%	17.5	65.8%	65.7%	0.2
Male sex, %	34.2%	42.7%	-17.5	34.2%	34.3%	-0.2
Race/ethnicity, % in category:						
Black, non-Hispanic	17.5%	10.0%	20.9	17.5%	17.3%	0.5
Hispanic	0.3%	1.5%	-15.6	0.3%	0.3%	-0.2
White, non-Hispanic	79.5%	84.9%	-13.6	79.5%	80.1%	-1.2
Other [e]	2.7%	3.7%	-5.8	2.7%	2.3%	1.8
Disabled, %	68.2%	68.8%	-1.3	68.2%	68.2%	0.1
Elixhauser Comorbidity Index in 2017	4.80	3.97	0.3	4.80	4.98	-0.04
Area Deprivation Index (ADI) in 2017, % in category:						
Low (quintiles 1, 2, and 3 of ADI)	37.1%	40.9%	-7.7	37.1%	37.8%	-1.2
High (quintiles 4 and 5 of ADI)	62.9%	59.1%	7.6	62.9%	62.2%	1.2
Urban residence, % in category:						
Rural	11.6%	20.3%	-25.1	11.6%	11.9%	-0.8
Urban	88.4%	79.7%	24.9	88.4%	88.1%	0.7
**Nursing home care:**						
Received more than 100 days of long-term nursing home care in 2017, %	0.2%	0.5%	-6.1	0.2%	0.6%	-6.3

[a] Covariates used to construct propensity score weights. We weighted the comparison cohort to resemble the integration cohort on baseline covariates in 2017. See methods for details of the propensity score weighting methodology. See the notes to Table 1 in the main manuscript for information about the covariates.
[b] Numbers of dual eligible individuals in the integration and comparison cohorts who lived in the community in 2017.
[c] Differences in means or proportions of each characteristic between the integration and comparison cohorts, divided by the deviation of the characteristic (pooled across both cohorts). Standardized mean differences less than 0.2 indicate good balance.

© 2023 Roberts ET et al. *JAMA Health Forum.*

eTable 5: Difference-in-differences estimates, stratified according whether dual eligible individuals in 2017 resided in a nursing home, lived in community and were enrolled in an HCBS waiver program, or lived in community and not in an HCBS waiver program

Outcome [a,b]	Mean of outcome in integration cohort in 2017	Differential change from baseline to… Year one of integration (2018)	Year two of integration (2019)	Year three of integration (2020)
		Difference-in-differences estimate (95% CI)		
**Stratum: resided in a nursing home in 2017** (n=328 in integration cohort; n=985 in comparison cohort)				
Receipt of home and community-based services (HCBS) (days/month) [c]	0.92	-0.31 (-0.81, 0.20)	0.21 (-0.56, 0.97)	0.34 (-0.51, 1.19)
Care management and coordination:				
Outpatient visits (count/month)	0.23	-0.06* (-0.12, 0.00)	-0.09** (-0.16, -0.02)	-0.08* (-0.15, 0.00)
Standardized 30-day fills of chronic disease medications (count/month)	4.05	-0.22 (-0.46, 0.02)	-0.41 (-0.85, 0.04)	-0.37 (-0.80, 0.06)
Hospital visit followed within 14 days by an outpatient visit (proportion)	0.18	0.02 (-0.05, 0.09)	0.09 (-0.01, 0.20)	0.00 (-0.08, 0.08)
Hospital and post-acute care use:				
Inpatient care (days/month)	0.50	0.12 (-0.04, 0.28)	-0.08 (-0.21, 0.05)	0.05 (-0.20, 0.31)
Inpatient admissions for ambulatory care-sensitive conditions (count/month)	0.02	0.00 (-0.01, 0.01)	0.00 (-0.01, 0.01)	0.01 (-0.01, 0.01)
Emergency department visits and observation stays (count/month)	0.09	-0.02 (-0.05, 0.01)	-0.01 (-0.04, 0.02)	-0.01 (-0.04, 0.02)
Post-acute skilled nursing facility care (days/month)	1.37	-0.20 (-0.48, 0.07)	-0.24 (-0.51, 0.03)	-0.31 (-0.69, 0.07)
Post-acute home health care (days/month)	6.26	2.22**** (1.56, 2.90)	1.31**** (0.60, 2.02)	1.67**** (0.83, 2.51)
Long-term nursing home use:				
Long-term nursing home care (days/month) [c,d]	24.4	0.17 (-0.92, 1.25)	-0.28 (-1.59, 1.03)	0.02 (-1.45, 1.49)

© 2023 Roberts ET et al. *JAMA Health Forum.*

**Stratum: lived in the community and enrolled in an HCBS waiver program in 2017** (n=988 in integration cohort; n=448 in comparison cohort)				
Receipt of home and community-based services (HCBS) (days/month) [c]	22.3	2.07****<br>(0.95, 3.17)	3.02****<br>(1.58, 4.46)	3.97****<br>(2.48, 5.46)
Care management and coordination:				
Outpatient visits (count/month)	0.92	-0.04<br>(-0.10, 0.02)	-0.09*<br>(-0.18, 0.00)	-0.13**<br>(-0.22, -0.03)
Standardized 30-day fills of chronic disease medications (count/month)	4.12	0.01<br>(-0.14, 0.16)	-0.03<br>(-0.26, 0.20)	-0.32*<br>(-0.65, 0.00)
Hospital visit followed within 14 days by an outpatient visit (proportion)	0.49	0.01<br>(-0.06, 0.08)	-0.03<br>(-0.11, 0.05)	-0.06<br>(-0.15, 0.03)
Hospital and post-acute care use:				
Inpatient care (days/month)	0.37	-0.05<br>(-0.19, 0.09)	-0.07<br>(-0.22, 0.08)	0.11<br>(-0.03, 0.25)
Inpatient admissions for ambulatory care-sensitive conditions (count/month)	0.01	0.00<br>(-0.01, 0.01)	0.00<br>(-0.01, 0.01)	0.00<br>(-0.01, 0.01)
Emergency department visits and observation stays (count/month)	0.15	0.02<br>(-0.10, 0.05)	0.01<br>(-0.02, 0.04)	0.00<br>(-0.03, 0.02)
Post-acute skilled nursing facility care (days/month)	0.57	0.04<br>(-0.14, 0.22)	-0.10<br>(-0.36, 0.17)	-0.23<br>(-0.49, 0.04)
Post-acute home health care (days/month)	2.79	-0.20<br>(-1.00, 0.60)	0.28<br>(-0.47, 1.03)	1.85****<br>(1.05, 2.65)
Long-term nursing home use:				
Long-term nursing home care (days/month) [c,d]	0.19	0.05<br>(-0.20, 0.30)	-0.59<br>(-1.39, 0.21)	-0.87<br>(-1.84, 0.11)
**Stratum: lived in the community and not in an HCBS waiver program in 2017** (n=6,651 in integration cohort; n=2,399 in comparison cohort)				
Receipt of home and community-based services (HCBS) (days/month) [c]	0.04	0.02<br>(-0.07, 0.11)	0.19*<br>(0.00, 0.38)	0.28**<br>(0.04, 0.52)
Care management and coordination:				
Outpatient visits (count/month)	0.73	-0.03<br>(-0.07, 0.01)	-0.04<br>(-0.09, 0.01)	-0.10**<br>(-0.15, -0.05)
Standardized 30-day fills of chronic disease medications (count/month)	3.15	0.05<br>(-0.01, 0.11)	0.02<br>(-0.05, 0.09)	0.07<br>(-0.01, 0.16)

© 2023 Roberts ET et al. *JAMA Health Forum.*

Hospital visit followed within 14 days by an outpatient visit (proportion)	0.48	-0.03 (-0.07, 0.01)	0.03 (-0.02, 0.08)	0.01 (-0.03, 0.05)
Hospital and post-acute care use:				
Inpatient care (days/month)	0.11	-0.02 (-0.07, 0.03)	-0.01 (-0.06, 0.04)	0.02 (-0.02, 0.06)
Inpatient admissions for ambulatory care-sensitive conditions (count/month)	0.004	0.00 (-0.01, 0.01)	0.00 (-0.01, 0.01)	0.01 (-0.01, 0.01)
Emergency department visits and observation stays (count/month)	0.09	0.00 (-0.01, 0.01)	-0.01 (-0.02, 0.00)	-0.01 (-0.02, 0.00)
Post-acute skilled nursing facility care (days/month)	0.12	0.03 (-0.01, 0.07)	0.04* (0.00, 0.08)	0.03 (-0.01, 0.07)
Post-acute home health care (days/month)	0.48	0.10 (-0.04, 0.23)	0.04 (-0.12, 0.20)	0.13* (-0.01, 0.27)
Long-term nursing home use:				
Long-term nursing home care (days/month) [c,d]	0.03	-0.01 (-0.08, 0.07)	0.00 (-0.15, 0.16)	-0.06 (-0.30, 0.19)

P-values denoted as follows: * P<0.1, ** P<0.05, *** P<0.01, **** P<0.001.

[a] Table reports difference-in-differences estimates, which are differential changes in outcome between the integration and comparison cohorts from baseline through three years of integration (2018, 2019, and 2020). The unit of analysis is the person-month. The baseline period is 2015-2017 for all outcomes except those measured from Medicaid claims, where the baseline period was limited to 2017 due to available data (outcomes indicated by footnote c). Estimates adjusted for covariates described in methods and weighted by propensity score weights based on covariates in Table 1 of the main manuscript. Estimates stratified according whether dual eligible individuals in 2017: 1) lived in a nursing home, 2) lived in the community and qualified for an HCBS waiver program, or 3) lived in the community and were ineligible for an HCBS waiver program.

[b] See the notes to Table 2 of the main manuscript for descriptions of the outcome variables.

[c] Measured from Medicaid claims.

[d] Due to differences in mortality rates across the three strata (see eFigure 5), over the post-integration period, a weighted average of these stratum-specific estimates becomes weighted *less* towards dual eligible individuals who were nursing home residents at baseline (among whom mortality was higher) and *more* towards dual eligible individuals who were living in the community at baseline (among whom mortality was lower). This compositional change in the weighted average explains why, in the overall difference-in-differences estimates reported in Table 2, we find a greater reduction in long-term nursing home stays in the *comparison* group than in the integration group (producing a positive and statistically significant difference-in-differences estimate) while stratum-specific estimates for this outcome are all small and statistically insignificant.

© 2023 Roberts ET et al. *JAMA Health Forum.*

eFigure 7: Monthly changes in long-term care eligibility among dual eligible individuals in the integration and comparison cohorts, 2018-2020

[A.1] Proportions reclassified as eligible for and receiving HCBS (community-dwelling and HCBS ineligible in 2017)
(Main sample, including 2018-20 decedents)

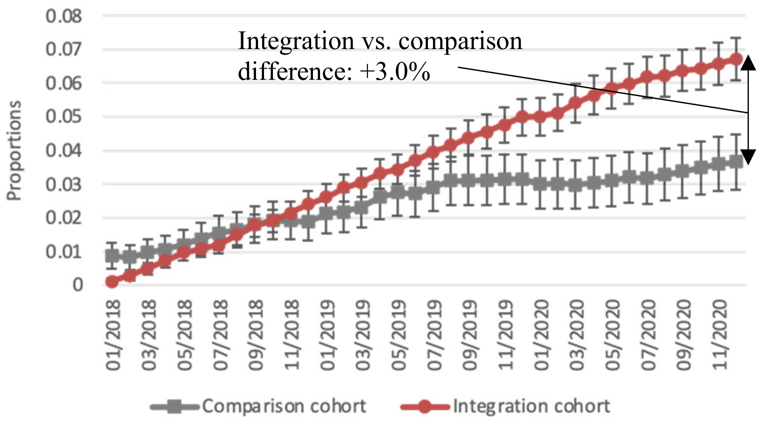

[A.2] Proportions reclassified as eligible for and receiving HCBS (community-dwelling and HCBS ineligible in 2017)
(Sensitivity analysis sample, excluding 2018-20 decedents)

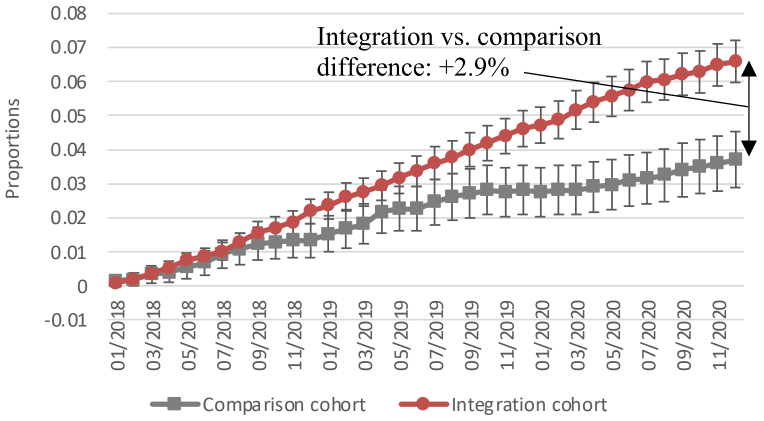

[B.1] Proportions remaining classified as eligible for and receiving HCBS (community-dwelling and HCBS eligible in 2017)
(Main sample, including 2018-20 decedents)

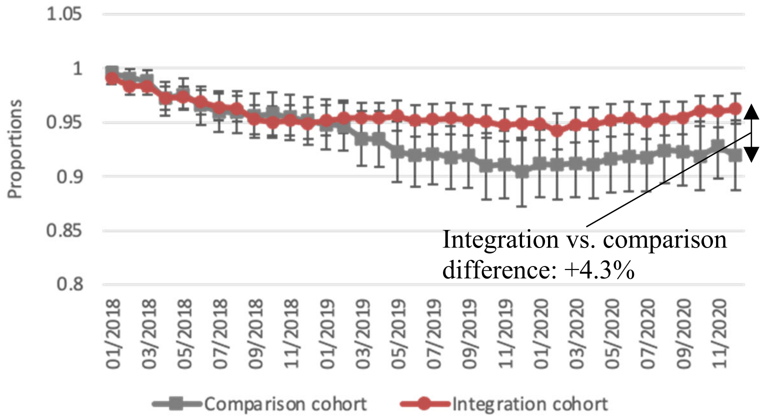

[B.2] Proportions remaining classified as eligible for and receiving HCBS (community-dwelling and HCBS eligible in 2017)
(Sensitivity analysis sample, excluding 2018-20 decedents)

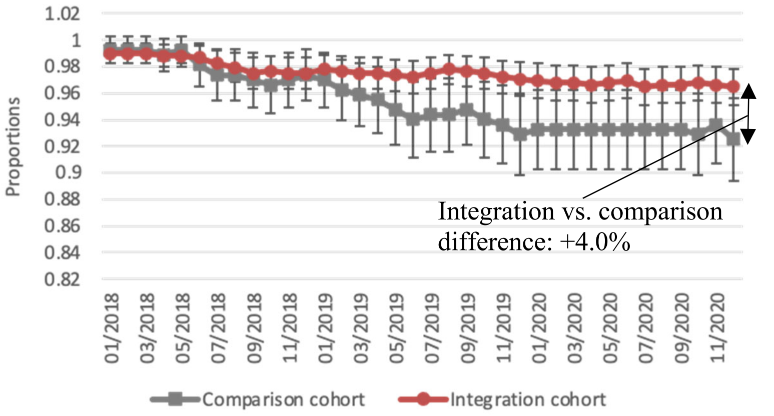

© 2023 Roberts ET et al. *JAMA Health Forum.*

[C.1] Proportions reclassified as nursing facility care-eligible (community-dwelling and HCBS eligible in 2017)

(Main sample, including 2018-20 decedents)

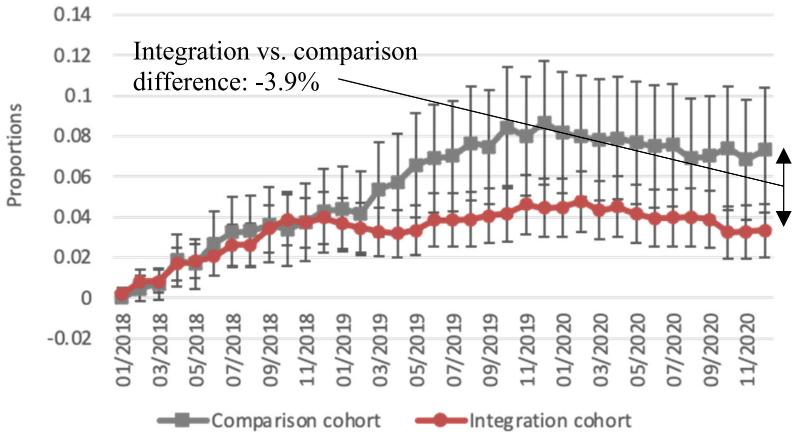

[C.2] Proportions reclassified as nursing facility care-eligible (community-dwelling and HCBS eligible in 2017)

(Sensitivity analysis sample, excluding 2018-20 decedents)

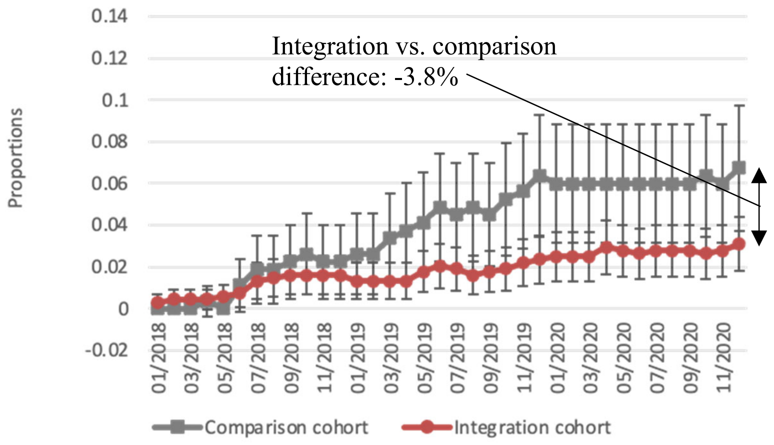

Graphs display changes in the integration and comparison cohorts from 2017 (baseline) through 2018-2020 in: 1) the proportions of community-dwelling dual eligible individuals who did not qualify for an HCBS waiver program in 2017 and were reclassified as HCBS-eligible from 2018-2020; 2) the proportions of community-dwelling dual eligible individuals who qualified for an HCBS waiver program in 2017 and remained HCBS-eligible from 2018-2020; and 3) the proportions of community-dwelling dual eligible individuals who qualified for an HCBS waiver program in 2017 and were re-classified as eligible for long-term nursing facility care from 2018-2020. Estimates based on model (3) in the online supplement. Plotted points are estimates and whiskers ae 95% confidence intervals. In our sample, 9,050 dual eligible individuals (76.7% of the total study sample) lived in the community and did not qualify for an HCBS waiver program in 2017 (n=6,651 in the integration cohort [83.5% of integration cohort members] and n=2,399 in the comparison cohort [62.6% of comparison cohort members]). In 2017, 1,436 dual eligible individuals (12.2% of the total study sample) qualified for an HCBS waiver program (n=988 in the integration cohort [12.4% of integration cohort members] and 448 in the comparison cohort [11.7% of comparison cohort members]).

© 2023 Roberts ET et al. *JAMA Health Forum.*

eReferences

1. SNP Comprehensive Report 2023 (April). 2023. Accessed May 1, 2023. https://www.cms.gov/research-statistics-data-and-systemsstatistics-trends-and-reportsmcradvpartdenroldataspecial-needs/snp-comprehensive-report-2023-04

2. Community Healthchoices Overview. 2019. https://www.dhs.pa.gov/HealthChoices/HC-Services/Documents/ProviderSummit-Overview.pptx. Accessed October 30, 2022.

3. Degenholtz HB, Kastner K, Bear T, Cole E, Yauch J. Evaluation of the Community Healthchoices (Chc) Program: Summary of Activities, Findings and Recommendations (2016 to 2019). 2021. January. https://www.dhs.pa.gov/HealthChoices/HC-Providers/Documents/CHC%20Public%20Report%20Jan%202021.pdf

4. 2022 Community Healthchoices Agreement. 2022. https://www.dhs.pa.gov/HealthChoices/HC-Providers/Documents/2022%20CHC%20Agreement.pdf

5. Community Health Choices: Coordination with Medicare. 2018. Accessed October 30, 2022. https://www.dhs.pa.gov/HealthChoices/HC-Services/Documents/Coordination%20of%20Medicare.pdf

6. Chapter 4: Establishing a Unified Program for Dually Eligible Beneficiaries: Design Considerations. 2021. March. Accessed March 21, 2022. https://www.macpac.gov/wp-content/uploads/2021/03/Chapter-4-Establishing-a-Unified-Program-for-Dually-Eligible-Beneficiaries-Design-Considerations.pdf

© 2023 Roberts ET et al. *JAMA Health Forum.*

7. Chapter 6: Improving Integration for Dually Eligible Beneficiaries: Strategies for State Contracts with Dual Eligible Special Needs Plans. 2021. June. Accessed March 21, 2022. https://www.macpac.gov/wp-content/uploads/2021/06/Chapter-6-Improving-Integration-for-Dually-Eligible-Beneficiaries-Strategies-for-State-Contracts-with-Dual-Eligible-Special.pdf

8. Social Security Administration. Compilation of the Social Security Laws: Payments to Medicare+Choice Organizations. https://www.ssa.gov/OP_Home/ssact/title18/1853.htm. Accessed August 15, 2023.

9. Kind AJ, Jencks S, Brock J, et al. Neighborhood Socioeconomic Disadvantage and 30-Day Rehospitalization: A Retrospective Cohort Study. *Ann Intern Med*. 2014;161(11):765-74. doi:10.7326/M13-2946

10. Data from: 2010 Rural-Urban Commuting Area Codes, Zip Code File.2020 update. *Washington, DC*.

11. Anderson WL, Long SK, Feng Z. Effects of Integrating Care for Medicare-Medicaid Dually Eligible Seniors in Minnesota. *J Aging Soc Policy*. 2020;32(1):31-54. doi:10.1080/08959420.2018.1485396

12. Keohane LM, Zhou Z, Stevenson DG. Aligning Medicaid and Medicare Advantage Managed Care Plans for Dual-Eligible Beneficiaries. *Med Care Res Rev*. 2022;79(2):207-217. doi:10.1177/10775587211018938

13. Wysocki A, Libersky J, Gellar J, Liu S, Pearse M. Medicaid 1115 Demonstration Evaluation Design Plan: Managed Long-Term Services and Supports. Report. 2019. January.

© 2023 Roberts ET et al. *JAMA Health Forum.*

14. Issues Affecting Dual-Eligible Beneficiaries—Cms's Financial Alignment Demonstration and the Medicare Savings Programs. *Report to the Congress: Medicare and the Health Care Delivery System*. Medicare Payment Advisory Commission; 2016:chap Chapter 9.

15. Greene AM, Feng Z. Massachusetts One Care Preliminary Fourth Evaluation Report. 2021.

16. Greene AM, Feng Z. Mycare Ohio Second Evaluation Report. 2022. March.

17. Kim H, Charlesworth CJ, McConnell KJ, Valentine JB, Grabowski DC. Comparing Care for Dual-Eligibles across Coverage Models: Empirical Evidence from Oregon. *Med Care Res Rev*. 2019;76(5):661-677. doi:10.1177/1077558717740206

18. Soper M, Kruse A, Dobson C. The Value of Pursuing Medicare-Medicaid Integration for Medicaid Agencies. 2019. November 22. Accessed October 31, 2022. http://www.advancingstates.org/hcbs/article/value-pursuing-medicare-medicaid-integration-medicaid-agencies

19. Feng Z, Wang J, Gadaska A, et al. Comparing Outcomes for Dual Eligible Beneficiaries in Integrated Care: Final Report. 2021. September. Accessed April 5, 2022. https://aspe.hhs.gov/sites/default/files/documents/9739cab65ad0221a66ebe45463d10d37/dual-eligible-beneficiaries-integrated-care.pdf

20. User Guide: Elixhauser Comorbidity Software Refined for Icd-10-Cm Diagnoses, V2022.1. 2021. October. Accessed June 1, 2022. https://www.hcup-us.ahrq.gov/toolssoftware/comorbidityicd10/CMR-User-Guide-v2022-1.pdf

© 2023 Roberts ET et al. *JAMA Health Forum.*

21. Dugoff EH, Schuler M, Stuart EA. Generalizing Observational Study Results: Applying Propensity Score Methods to Complex Surveys. *Health services research*. 2014;49(1):284-303. doi:10.1111/1475-6773.12090

22. Daw JR, Hatfield LA. Matching and Regression to the Mean in Difference-in-Differences Analysis. *Health services research*. 2018;53(6):4138-4156.

23. Roberts ET, McWilliams JM, Hatfield LA, et al. Changes in Health Care Use Associated with the Introduction of Hospital Global Budgets in Maryland. *JAMA Internal Medicine*. 2018;178(2):260-268. doi:10.1001/jamainternmed.2017.7455

© 2023 Roberts ET et al. *JAMA Health Forum.*
