# Supplementary material for: Changes in Care Associated With Integrating Medicare and Medicaid for Dual-Eligible Individuals
Source: JAMA Health Forum. 2023 Dec 21;4(12):e234583. doi: 10.1001/jamahealthforum.2023.4583 (PMC10739174; doi:10.1001/jamahealthforum.2023.4583)
Supplement: Supplement 2. — Data Sharing Statement [file jamahealthforum-e234583-s002.pdf]

Data Sharing Statement

Roberts. Changes in Care Associated With Integrating Medicare and Medicaid for Dual-Eligible Individuals. *JAMA Health Forum*. Published December 21, 2023.
doi:10.1001/jamahealthforum.2023.4583

Data
Data available: No

Additional Information
Explanation for why data not available: We are unable to share individual patient data due to prohibitions of our Data Use Agreements with data providers. We will make a codebook and full analytic code publicly available on a public repository (e.g., Figshare) along with a readme file to orient users to this documentation. We have enclosed the following statement in the acknowledgements: Analytic code for this study has been posted on Figshare, an online repository (DOI: 10.6084/m9.figshare.24224284), including a directory and readme files to orient users to the analytic code
